# Prognostic value of oxygen saturation index trajectory phenotypes on ICU mortality in mechanically ventilated patients: a multi-database retrospective cohort study

**DOI:** 10.1186/s40560-023-00707-x

**Published:** 2023-11-29

**Authors:** Xiawei Shi, Yangyang Shi, Liming Fan, Jia Yang, Hao Chen, Kaiwen Ni, Junchao Yang

**Affiliations:** 1 Zhejiang Chinese Medical University, Hangzhou, China; 2 School of Chinese Medicine, Hong Kong Baptist University, Hong Kong, China; 3 The First Affiliated Hospital of Zhejiang Chinese Medical University, No. 54 Youdian Road, Shangcheng District, Hangzhou, 310006 Zhejiang China; 4 Longhua Hospital, Shanghai University of Traditional Chinese Medicine, Shanghai, China

**Keywords:** Oxygen saturation index, Invasive mechanical ventilation, Intensive care unit, Mortality

## Abstract

**Background:**

Heterogeneity among critically ill patients undergoing invasive mechanical ventilation (IMV) treatment could result in high mortality rates. Currently, there are no well-established indicators to help identify patients with a poor prognosis in advance, which limits physicians’ ability to provide personalized treatment. This study aimed to investigate the association of oxygen saturation index (OSI) trajectory phenotypes with intensive care unit (ICU) mortality and ventilation-free days (VFDs) from a dynamic and longitudinal perspective.

**Methods:**

A group-based trajectory model was used to identify the OSI-trajectory phenotypes. Associations between the OSI-trajectory phenotypes and ICU mortality were analyzed using doubly robust analyses. Then, a predictive model was constructed to distinguish patients with poor prognosis phenotypes.

**Results:**

Four OSI-trajectory phenotypes were identified in 3378 patients: low-level stable, ascending, descending, and high-level stable. Patients with the high-level stable phenotype had the highest mortality and fewest VFDs. The doubly robust estimation, after adjusting for unbalanced covariates in a model using the XGBoost method for generating propensity scores, revealed that both high-level stable and ascending phenotypes were associated with higher mortality rates (odds ratio [OR]: 1.422, 95% confidence interval [CI] 1.246–1.623; OR: 1.097, 95% CI 1.027–1.172, respectively), while the descending phenotype showed similar ICU mortality rates to the low-level stable phenotype (odds ratio [OR] 0.986, 95% confidence interval [CI] 0.940–1.035). The predictive model could help identify patients with ascending or high-level stable phenotypes at an early stage (area under the curve [AUC] in the training dataset: 0.851 [0.827–0.875]; AUC in the validation dataset: 0.743 [0.709–0.777]).

**Conclusions:**

Dynamic OSI-trajectory phenotypes were closely related to the mortality of ICU patients requiring IMV treatment and might be a useful prognostic indicator in critically ill patients.

**Supplementary Information:**

The online version contains supplementary material available at 10.1186/s40560-023-00707-x.

## Background

Invasive mechanical ventilation (IMV) is a cornerstone of intensive care medicine and a life-saving tool utilized worldwide to manage various conditions, from heart failure to respiratory failure [1, 2]. A national population-based epidemiological study revealed that IMV utilization for nonsurgical purposes has increased since 1993, with the rate peaking at 310.9 per 100,000 adults in 2009 in the United States of America [3]. Nevertheless, IMV-associated hospital mortality rates remain high, ranging from 21 to 38% [4–6].

Theoretically, uniform management strategies may fail and increase mortality rate in the case of heterogeneity. However, few studies have focused on the heterogeneity of patients with critical illnesses requiring IMV treatment. Su et al. found that five novel phenotypes were associated with mortality in patients with critical illness and IMV treatment using machine learning [7]. The process of identifying different phenotypes necessitates the gathering of data from 22 variables upon admission, a procedure that is obviously time-consuming. Moreover, critically ill patients’ conditions are prone to sudden changes [8]. As a result, the application of these five novel phenotypes presents considerable challenges in clinical practice.

Driving pressure and mechanical power were significant predictors of mortality in adult patients requiring mechanical ventilation [9, 10]. However, a large observational study showed that plateau pressure was only measured in 40.1% of patients with acute respiratory distress syndrome (ARDS), let alone the driving pressure and mechanical power that require the measurement of plateau pressure [11]. This may not have been included because calculating the plateau pressure requires patients to be fully sedated with an inspiratory pause time of 2 s, which makes frequent measurement of the plateau pressure inconvenient. Moreover, it may cause patient–ventilator asynchrony [12]. The oxygen index (OI), a composite index integrating the mean airway pressure (MAP), the fraction of inspired O_2_ (FiO_2_) and partial pressure of O_2_ (PaO_2_), is an important predictor of mortality in pediatric and adult patients undergoing IMV treatment [13, 14]. As obtaining PaO_2_ in the blood requires invasive arterial blood gas analysis, the measurement of OI cannot be conducted freely and repeatedly. Therefore, identifying a new noninvasive and user-friendly indicator is crucial for patients requiring IMV treatment.

The oxygen saturation index (OSI) is a noninvasive prognostic indicator as effective as OI and is mostly used in pediatrics or newborns with hypoxemic respiratory failure or acute lung injury [15–18]. Calculated using the MAP, FiO_2_, and pulse oximetry (SpO_2_), it could reflect the oxygenation status and airway resistance. Several studies have used the OSI value in assessing adult patients with ARDS [19, 20]. Nevertheless, these studies typically included the OSI value at admission, neglecting to dynamically monitor changes in OSI values. In fact, compared to relying solely on static data, long-term monitoring of prognostic indicators may provide more meaningful insights into revealing heterogeneity and promoting the timely adjustment of management strategies [21]. Moreover, the prognostic value of OSI was never explored in the patients with IMV treatment. Therefore, this study aimed to explore the association between the dynamic OSI trajectories and ICU mortality in adult patients undergoing IMV treatment to provide a theoretical basis for the improvement of current treatment strategies.

## Methods

### Data source and study population

This retrospective cohort study enrolled patients who were admitted from multiple critical care units from 2008 to 2019. The data were obtained from two databases: the Medical Information Market for Intensive Care IV (MIMIC-IV) and the eICU Collaborative Research Database (eICU-CRD) [22, 23]. The authors completed a “protecting human subjects” training and received approval from the Institutional Review Boards of the Massachusetts Institute of Technology prior to data retrieval. This study was approved by the Ethics Committee of the First Affiliated Hospital of Zhejiang Chinese Medical University (No. 2023-KLS-173-01). Due to the retrospective design, the requirement of informed consent for patients was waived. Data were extracted using PostGreSQL tools (v9.6; The PostGreSQL Global Development Group, USA, http://www.postgresql.org) by S.X., who completed a training program at the National Institutes of Health (Certificate Number: 53248857).

Patients who met any of the following criteria were excluded from the study: (1) age < 18 years; (2) ICU length of stay < 5 days; (3) missing MAP, FiO_2_, or SpO_2_ values within the first 5 days after ICU admission (needed for the group-based trajectory model [GBTM] analysis); and (4) IMV treatment < 5 days. The study enrolled 3378 patients, including 1854 and 1524 patients from the MIMIC-IV and eICU-CRD databases, respectively.

### Patient characteristics and outcomes

The following information was collected upon admission: (1) demographic data: age, gender, and ethnicity; (2) physical examination findings: body mass index (BMI), respiratory rate, and heart rate; (3) ventilator setting: positive end expiratory pressure (PEEP), plateau pressure, tidal volume, MAP, FiO_2_, and SpO_2_; (4) laboratory events: PaO_2_, PaCO_2_, PH, white blood cell count, hemoglobin, and platelet count; (5) comorbidity diseases: heart failure (HF), chronic kidney disease (CKD), acute kidney injury (AKI), chronic obstructive pulmonary disease (COPD), malignancy, diabetes, and ARDS; (6) treatment: vasopressor therapy, dialysis, and neuromuscular blockades (NMBAs); and (7) clinical outcomes: acute physiology III score (APS-III), Sequential Organ Failure Assessment score (SOFA), ICU mortality, ventilation-free days (VFDs), and 21-day ICU mortality. Data were collected within 48 h of ICU admission, with only the first recorded admission eligible for analysis if a patient had multiple records. MAP, FiO_2_, and SpO_2_ were continuously collected every 6 h for 5 days, starting from ICU admission. The OSI was calculated using the following formula:

$$\text{OSI}=\frac{\text{Fi}{\text{O}}_{2}*\text{MAP}*100}{\text{Sp}{\text{O}}_{2}}.$$

The primary endpoint of this study was ICU mortality while VFDs and 21-day ICU mortality were secondary outcomes.

### Management of missing data

Missing data were common in large-scale medical databases. Covariates with over 15% missing data were removed to enhance the integrity of the data, reduce potential bias, and strengthen statistical power [24–27], such as mechanical power, the Oxford Acute Severity of Illness Score and chronic health points (Additional file 1: Fig. S1). For variables with the missing percentage less than 15%, multiple imputation method was utilized in our study. This method has been extensively utilized as the standard approach for handling missing data and could relatively precisely estimate correlations based on accessible data [28–31]. Briefly, ten datasets were generated to substitute the missing data utilizing a technique known as multiple imputation by chained equations (MICE) [32]. MICE applied distinct methods for different kinds of covariables. Linear regression was used for scale covariables and logistic regression was used for nominal covariables. Subsequently, each dataset estimated the odds ratio and variance‒covariance matrix, respectively. Ultimately, following Rubin’s rules [33], the ten datasets were merged into a comprehensive dataset to impute the missing data.

### Group-based trajectory model

A GBTM method was applied to identify distinct phenotypes that followed particular trajectories using the “traj” plugin in Stata software to investigate the dynamic OSI trajectories [34]. As critically ill patients on mechanical ventilation for ≤ 4 days are considered to be in the acute phase of mechanical ventilation, the OSI was recorded continuously for 5 days after ICU admission for further analysis [35]. The following parameters were used to determine the appropriate trajectories: Bayesian information criterion (BIC), -2*log-likelihood, average posterior probability, entropy, and the number of participants within each group. The OSI trajectories were initialized as quadratic shapes to determine the appropriate number of groups. Statistical models with two, three, or four classes were compared based on their respective BIC, -2*log-likelihood, and entropy. Models with an average posterior probability of < 0.7 and groups containing fewer than 5% of participants were excluded from subsequent analyses. The final functional form of the model was determined by evaluating three functional forms starting with the highest polynomial, including cubic, quadratic, and linear terms.

### Directed acyclic graphs

The directed acyclic graphs (DAG) method (Additional file 2: Fig. S2) was used to identify the potential covariates that need to be adjusted in the further study by the DAGitty tool (https://dagitty.net) [36–38]. We identified 21 covariates for further analysis based on literature review, our priori knowledge and the minimally sufficient adjustment set, including demographics [39, 40] (age, gender and ethnicity), BMI [41], PaCO_2_ [42], hemoglobin [43], comorbidities (ARDS, COPD, CKD, AKI, malignancy, HF and diabetes) [44–48], ventilation parameters (PEEP, plateau pressure, tidal volume, OSI at baseline) [49], APS-III score and treatment (dialysis, NMBAs and vasopressor therapy) [50–52].

### Doubly robust estimation

The doubly robust (DR) estimation method was useful for estimating the casual interference between variables and has been widely adopted in various studies for estimating the causal effect [53–55]. The DR estimation method combines two causal estimators: namely, the outcome regression and the exposure model based on propensity score. This advantageous approach ensures that even if only one of the two models is correctly specified, the causal estimation remains unbiased [56, 57]. In the present study, DR estimation was conducted to explore the associations between OSI-based phenotypes and ICU mortality following two steps. First, 21 covariables were included in multinomial logistic regression and Extreme Gradient Boosting (XGBoost) to generate the corresponding propensity scores, respectively. These propensity scores were then used to reweight the phenotypes and create balanced phenotypes using inverse probability of treatment weighting (IPTW). Covariates for balance were assessed by calculating the standardized mean difference (SMD) in effect size. Covariates with an SMD > 0.1 were considered imbalanced. Second, a weighted logistic regression based on the weighted model was conducted, thus completing the DR estimation. The variance inflation factor was tested for each multinomial model to detect multicollinearity. Generally, a variance inflation factor of > 5 is considered an indication of multicollinearity.

### Statistical analysis

Continuous variables were presented as the mean with standard deviation for normally distributed data and as the median with 25th and 75th percentiles for skewed data. Student’s *t* test (for normally distributed data) or the Wilcoxon–Mann–Whitney rank sum test (for skewed distributions) was used to determine statistical significance. Categorical variables were presented as frequencies and percentages, and the Chi-squared test was used to compare all phenotypes.

Kaplan–Meier analysis was used to examine the prognostic value of the OSI phenotypes for 21-day ICU mortality; to enhance its clinical utility, we developed a predictive model to identify patients with phenotypes associated with poor prognoses at an earlier stage. The training and validation groups were obtained from the MIMIC-IV and eICU-CRD databases, respectively. The synthetic minority oversampling technique (SMOTE) algorithm was used to equalize the number of groups in the training set [58]. The least absolute shrinkage and selection operator (LASSO) was used to screen for potential covariates, and a nomogram was created to show the results of the multinomial logistic regression. The area under the curve (AUC) was calculated using the receiver operating characteristic curve to evaluate the predictive power of the model. To evaluate the consistency between predicted and observed risks, a calibration curve and the Hosmer–Lemeshow test were conducted for the model. A decision curve analysis was used to assess the clinical usefulness of the model and to examine the net benefit.

Causal mediation analysis was employed to explore whether ARDS, as a potential factor affecting ICU mortality, could mediate the effects of the OSI phenotypes on ICU mortality [59, 60]. The total effect was separated into two parts: the average direct effect (ADE) and the average causal mediation effect (ACME). ADE represented the direct effect of the OSI phenotypes on ICU mortality, and ACME represented the indirect effect of the OSI phenotypes on ICU mortality due to ARDS. The predicted marginal effects of the OSI phenotypes were also estimated for ARDS.

Subgroup analysis with interaction effects was performed to investigate whether the effects of the OSI phenotypes on outcomes differed in specific populations. Sensitivity analysis was performed to validate the association between OSI-trajectory phenotypes and ICU mortality using the MIMIC-IV and eICU databases separately. A two-tailed test was performed, and *P* values < 0.05 were considered statistically significant. Stata software (v17.0; Stata Corporation, College Station, TX, USA), R (v4.2.1; http://www.R-project.org) and Python (v3.7.3; Python Software Foundation) were used to conduct statistical analyses.

## Results

### The OSI trajectories and baseline characteristics

**Fig. 1:**
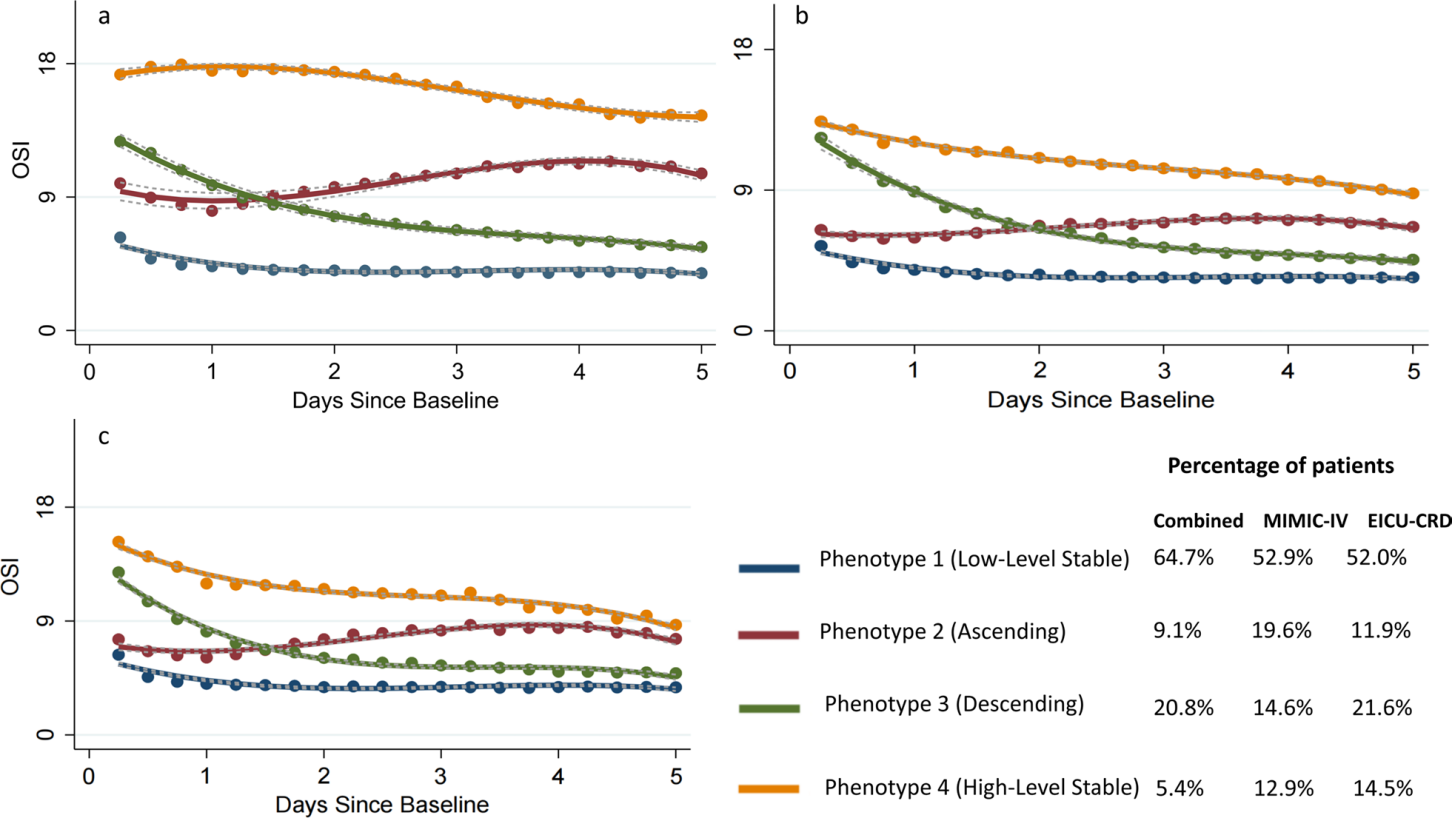
The OSI-trajectory phenotypes of patients with IMV treatment in ICU. As a finite mixture model, group-based trajectory model (GBTM) could identify the distinct phenotypes that follow particular trajectories. In this work, based on the OSI trajectories, four distinct phenotypes were identified: phenotype 1 (low-level stable), in which the OSI remained consistently stable at low levels; phenotype 2 (ascending), in which the OSI gradually increased over time; phenotype 3 (descending), in which the OSI gradually decreased over time; and phenotype 4 (high-level stable), in which the OSI remained consistently stable at high levels. This trend has been observed in the MIMIC-IV dataset, EICU-CRD dataset, as well as in the combined dataset of the two databases. Phenotype 4 had almost 1.4 times higher odds ratio of mortality compared to the phenotype 1 in the doubly robust estimation when controlling unbalanced covariates. **a** The OSI-trajectory phenotypes in the combined dataset. **b** The OSI-trajectory phenotypes in MIMIC-IV dataset. **c** The OSI-trajectory phenotypes in EICU-CRD dataset. OSI: oxygen saturation index; ICU: intensive care medicine; ARDS: acute respiratory distress syndrome; APS-III score: Acute Physiology Score III; MIMIC-IV: the Medical Information Market for Intensive Care IV; EICU-CRD: the eICU Collaborative Research Database

A total of 25,679 adult patients with ICU lengths of stay greater than 5 days were gathered from two distinct databases. 22,301 patients were consequently excluded due to missing OSI values and IMV treatment for less than 5 days. Finally, this study enrolled 3378 patients who met the patient selection criteria (Additional file 3: Fig. S3). The phenotype selection process of the trajectory model is shown in Additional file 4: Tables S1 and S2. Based on the OSI trajectories, four distinct phenotypes were identified: phenotype 1 (low-level stable), in which the OSI remained consistently stable at low levels; phenotype 2 (ascending), in which the OSI gradually increased over time; phenotype 3 (descending), in which the OSI gradually decreased over time; and phenotype 4 (high-level stable), in which the OSI remained consistently stable at high levels (Fig. 1a). Similar phenotypes were observed in the MIMIC-IV and eICU-CRD databases (Fig. 1b and c).

Demographic data, vital signs, laboratory events, comorbidities, treatments, and clinical outcomes were compared among the four groups. Patients with the high-level stable phenotype were found to be the youngest among the four phenotypes, with the highest baseline BMI, OSI at baseline, APS-III score and sequential organ failure assessment (SOFA) score, ICU mortality (40.98%) and fewest VFDs (1.97 days) compared with the other phenotypes (Table 1).

**Table 1:** Comparison of baseline characteristics among four phenotypes

	Overall (*n* = 3378)	Phenotype 1 (*n* = 2194)	Phenotype 2 (*n* = 301)	Phenotype 3 (*n* = 700)	Phenotype 4 (*n* = 183)	*P*
Demographic data						
Age (years)	62.00 [50.00, 72.00]	63.00 [51.00, 74.00]	56.00 [46.00, 68.00]	60.00 [50.00, 71.00]	54.00 [43.00, 63.00]	< 0.001
Male, *n* (%)	1926 (57.02)	1217 (55.47)	187 (62.13)	412 (58.86)	110 (60.11)	0.071
Ethnicity, *n* (%)						0.821
Black	368 (10.89)	25 (8.31)	243 (11.08)	77 (11.00)	23 (12.57)	
White	2255 (66.76)	206 (68.44)	1459 (66.50)	471 (67.29)	119 (65.03)	
Other	755 (22.35)	70 (23.26)	492 (22.42)	152 (21.71)	41 (22.40)	
Physical examination findings						
BMI	28.55 [24.13, 34.69]	27.43 [23.42, 32.45]	31.19 [25.90, 38.15]	30.35 [25.16, 37.15]	35.19 [28.31, 45.31]	< 0.001
Respiratory rate (beats/min)	20.00 [16.00, 24.00]	19.00 [16.00, 23.00]	20.00 [16.00, 24.00]	22.00 [17.75, 26.00]	22.00 [18.00, 28.00]	< 0.001
Heart rate (beats/min)	93.00 [78.00, 109.00]	91.00 [77.00, 106.00]	96.00 [83.00, 110.00]	98.00 [81.00, 116.00]	102.00 [85.50, 120.00]	< 0.001
Ventilator setting						
PEEP (cmH_2_O)	5.00 [5.00, 8.00]	5.00 [5.00, 6.00]	5.86 [5.00, 10.00]	8.00 [5.00, 10.00]	10.00 [5.48, 12.00]	< 0.001
Plateau pressure (cmH_2_O)	20.00 [16.00, 24.00]	18.00 [15.00, 21.58]	22.00 [18.00, 26.00]	24.00 [20.00, 27.00]	27.00 [23.00, 32.00]	< 0.001
Tidal volume (ml)	480.00 [400.25, 525.00]	480.65 [402.00, 522.00]	500.00 [440.00, 550.00]	465.50 [400.00, 517.75]	455.00 [400.00, 529.50]	0.047
OSI at baseline	7.00 [4.50, 11.17]	5.22 [4.00, 8.00]	9.38 [6.12, 12.50	12.00 [8.59, 16.10]	17.07 [12.18, 21.00]	< 0.001
Lab events						
PaO_2_ (mmHg)	117.37 [80.00, 194.31]	137.00 [91.00, 216.00]	99.00 [72.00, 168.00]	91.00 [68.00, 143.25]	78.00 [61.50, 106.00]	< 0.001
PaCO_2_(mmHg)	42.00 [35.52, 50.00]	39.93 [34.00, 47.00]	45.00 [38.00, 53.00]	46.00 [39.00, 55.85]	49.00 [41.00, 60.00]	< 0.001
PH	7.34 [7.26, 7.41]	7.36 [7.28, 7.43]	7.32 [7.25, 7.38]	7.29 [7.21, 7.37]	7.29 [7.19, 7.36]	< 0.001
WBC (*10^9^/L)	12.70 [9.10, 17.50]	12.60 [9.12, 17.10]	12.40 [8.40, 17.80]	13.15 [9.10, 18.92]	13.80 [9.55, 17.80]	0.085
HB (g/L)	10.80 [9.10, 12.60]	10.70 [9.00, 12.50]	11.10 [9.30, 12.90]	11.10 [9.30, 12.80]	11.10 [9.40, 12.75]	0.001
PLT (*10^9^/L)	195.00 [141.00, 259.00]	195.00 [143.00, 259.00]	192.00 [133.00, 244.00]	198.00 [136.00, 273.50]	198.00 [135.00, 249.00]	0.241
Comorbidity disease, *n* (%)						
Heart failure	661 (19.57)	413 (18.82)	56 (18.60)	154 (22.00)	38 (20.77)	0.290
Chronic kidney disease	239 (7.08)	164 (7.47)	17 (5.65)	45 (6.43)	13 (7.10)	0.591
Acute kidney injury	1564 (46.30)	912 (41.57)	161 (53.49)	379 (54.14)	112 (61.20)	< 0.001
Malignancy	227 (6.72)	148 (6.75)	16 (5.32)	55 (7.86)	8 (4.37)	0.261
COPD	709 (20.99)	432 (19.69)	73 (24.25)	161 (23.00)	43 (23.50)	0.087
Diabetes	814 (24.10)	529 (24.11)	58 (19.27)	177 (25.29)	50 (27.32)	0.144
ARDS	2039 (60.36)	1205 (54.92)	211 (70.10)	482 (68.86)	141 (77.05)	< 0.001
Treatment, *n* (%)						
Vasopressor therapy	1777 (52.61)	965 (43.98)	199 (66.11)	478 (68.29)	135 (73.77)	< 0.001
Dialysis	518 (15.33)	257 (11.71)	64 (21.26)	134 (19.14)	63 (34.43)	< 0.001
NMBAs	619 (18.32)	259 (11.80)	107 (35.55)	169 (24.14)	84 (45.90)	< 0.001
Clinical outcomes						
APS-III score	74.00 [56.00, 95.00]	71.00 [51.25, 90.00]	79.00 [62.00, 97.00]	79.00 [61.00, 100.00]	97.00 [69.00, 119.00]	< 0.001
SOFA score	5.00 [1.00, 8.00]	4.00 [1.00, 7.00]	5.00 [2.00, 9.00]	6.00 [2.00, 9.00]	6.00 [3.00, 10.00]	< 0.001
ICU mortality	773 (22.88)	454 (20.69)	95 (31.56)	149 (21.29)	75 (40.98)	< 0.001
VFDs	3.76 [0.40, 8.02]	4.24 [0.52, 8.21]	2.90 [0.25, 7.58]	3.46 [0.44, 7.58]	1.97 [0.21, 7.06]	0.004

BMI: body mass index; OSI: oxygen saturation index; WBC: white blood cell; HB: hemoglobin; PLT: platelet; COPD; chronic obstructive pulmonary disease; NMBAs: neuromuscular blockades; ARDS: acute respiratory distress syndrome; APS-III score: Acute Physiology III score; SOFA score: Sequential Organ Failure Assessment score; ICU: intensive care unit; VFDs: ventilation-free days

### Associations between the OSI trajectories and ICU mortality

**Fig. 2:**
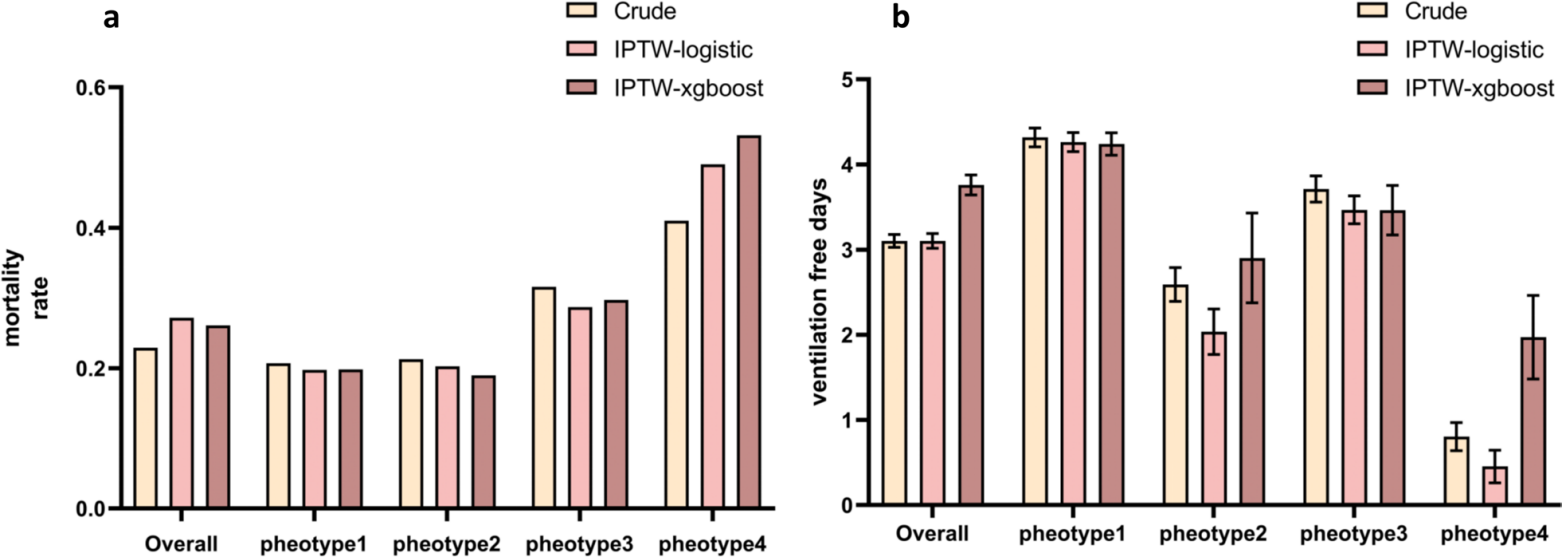
The comparison of ICU mortality (**a**) and ventilation-free days (**b**) among four phenotypes. Patients with phenotype 4 exhibited the highest mortality rate and fewest VFDs compared to patients with the other phenotypes, regardless of the use of the crude dataset, IPTW based on the logistic regression dataset, or IPTW based on the XGBoost dataset. VFDs: ventilation-free days; IPTW: inverse probability of treatment weighting; XGBoost: Extreme Gradient Boosting

SMDs generated using the IPTW method are presented in Additional file 5: Fig. S4. The SMD generated using XGBoost was less than that generated using the logistic regression method. The majority of the covariates were evenly balanced, except for age, plateau pressure, OSI at baseline, APS-III score, PEEP, PaCO_2_, vasopressor therapy, and NMBAs, when IPTW was used based on multinomial logistic regression or XGBoost. Patients with the high-level stable phenotype exhibited the highest mortality rate and fewest VFDs compared with patients with the other phenotypes, regardless of the crude dataset, IPTW based on the logistic regression dataset, or IPTW based on the XGBoost dataset (Fig. 2). Similar results were obtained for the MIMIC-IV and eICU-CRD datasets, demonstrating the robustness of the findings (Additional file 6: Fig. S5).

DR estimation showed that the high-level stable and ascending phenotypes were associated with significantly higher mortality risks compared with the low-level stable phenotype. Furthermore, no differences were observed between the low-level stable and descending phenotypes in the logistic (OR_ascending_: 1.522, 95% CI 1.189–1.948; OR_descending_: 1.069, 95% CI 0.849–1.347; OR_High-Level Stable_: 2.877, 95% CI 2.099–3.945) and the XGBoost (OR_ascending_: 1.097, 95% CI 1.027–1.172; OR_descending_: 0.986, 95% CI 0.940–1.035; OR_High-Level_: 1.422, 95% CI 1.246–1.623) models adjusted for the unbalanced covariates. These results were consistent in the models with univariate or all covariates adjusted, regardless of whether the IPTW method was constructed using logistic regression or the XGBoost method, indicating the robustness of the findings (Table 2). In addition, similar results were also observed in the MIMIC-IV and eICU-CRD databases in the sensitivity analysis (Additional file 4: Table S3).

**Table 2:** Results of doubly robust estimation

	Low-level stable	Ascending		Descending		High-level stable	
		OR (95% CI)	*P* value	OR (95% CI)	*P* value	OR(95%CI)	*P* value
IPTW-logistic							
Propensity score IPTW	Reference	1.430 (1.118–1.830)	0.004	1.020(0.813–1.278)	0.866	2.483(1.859–3.317)	< 0.001
Doubly robust with unbalanced covariables^a^	Reference	1.522(1.189–1.948)	< 0.001	1.069(0.849–1.347)	0.568	2.877(2.099–3.945)	< 0.001
Doubly robust with all covariables^b^	Reference	1.473(1.152–1.884)	0.002	1.019(0.817–1.270)	0.869	2.545(1.893–3.422)	< 0.001
IPTW-XGBoost							
Propensity score IPTW	Reference	1.532(1.200–1.956)	0.001	0.977(0.771–1.238)	0.676	2.988(2.144–4.165)	< 0.001
Doubly robust with unbalanced covariables^a^	Reference	1.097(1.027–1.172)	< 0.001	0.986(0.940–1.035)	0.849	1.422(1.246–1.623)	< 0.001
Doubly robust with all covariables^b^	Reference	1.500(1.178–1.911)	0.001	0.936(0.750–1.168)	0.556	2.776(1.988–3.875)	< 0.001

^a^Adjusted for plateau pressure, OSI at baseline, PEEP, NMBAs, APS-III score, PaCO_2_, vasopressor therapy, age

^b^Adjusted for plateau pressure, OSI at baseline, PEEP, NMBAs, APS-III score, PaCO_2_, vasopressor therapy, age, dialysis therapy, ARDS, AKI, BMI, gender, diabetes, tidal volume, ethnicity, HB, COPD, malignancy, HF, CKD

IPTW: inverse probability of treatment weighting; OR: odds ratio; CI: confidence Interval; OSI: oxygen saturation index; NMBAs: neuromuscular blockades; ARDS: acute respiratory distress syndrome; APS-III score: acute physiology III score; AKI: acute kidney injury; BMI: body mass index; HB: hemoglobin; COPD: chronic obstructive pulmonary disease; HF: heart failure; CKD: chronic kidney disease

### Prognostic value of OSI trajectories for 21-day ICU mortality

**Fig. 3:**
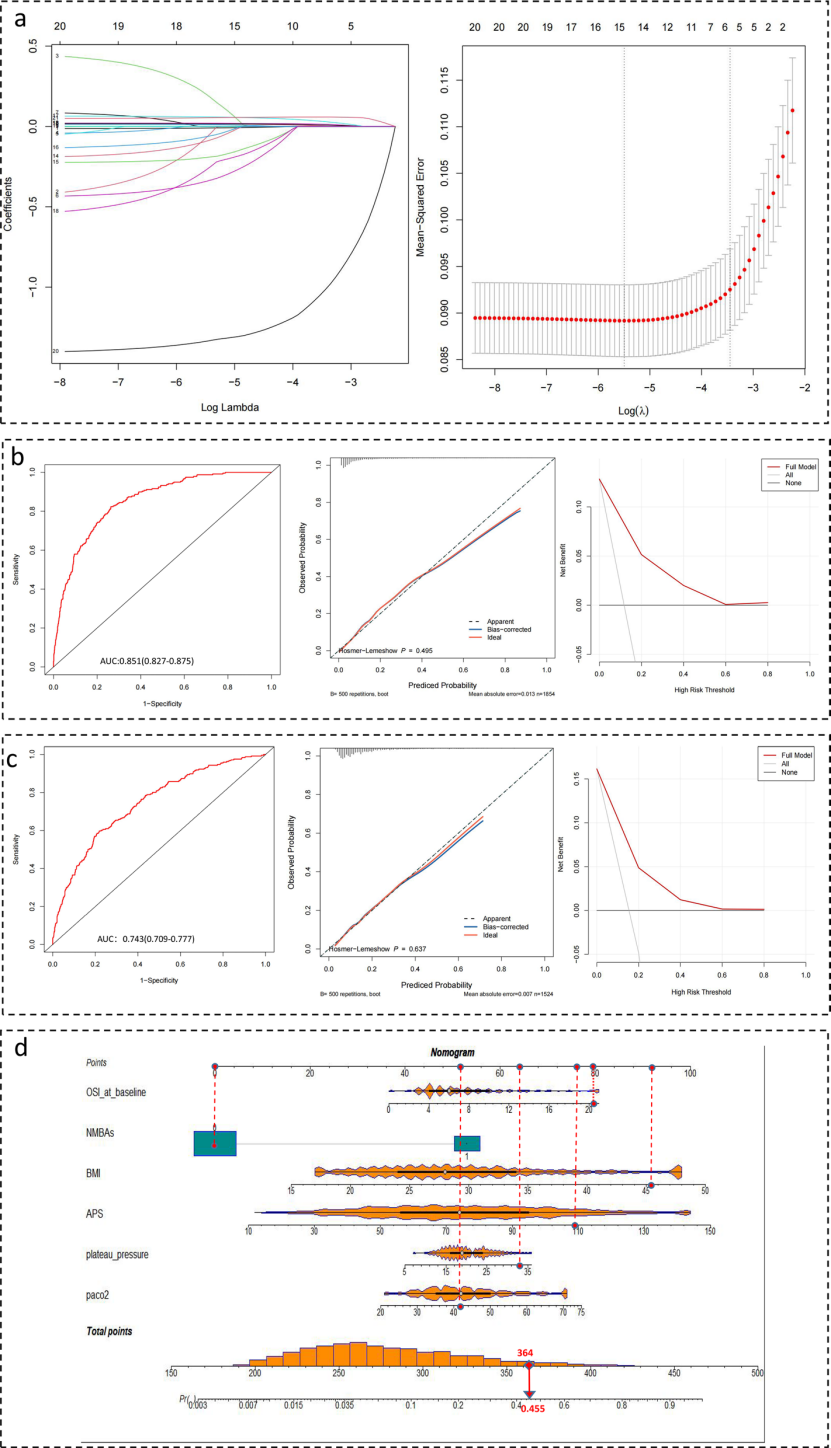
The early predictive model to identify the OSI-trajectory phenotypes related to the different prognoses. **a** Six distinctive characteristics of patients with worse prognosis (the high-level stable and ascending phenotypes) and better prognosis (the descending and low-level stable phenotypes) were selected using LASSO regression analysis. **b**, **c** MIMIC-IV and EICU-CRD database was used as training and validation group, respectively. The ROC curve, calibration curve and DCA analysis demonstrated that the predictive model exhibited strong ability of discrimination, calibration and clinical utilization. **d** The nomogram was plotted to better visualize our predictive model. OSI: oxygen saturation index; LASSO: The Least Absolute Shrinkage and Selection Operator; MIMIC-IV: the Medical Information Market for Intensive Care IV; EICU-CRD: the eICU Collaborative Research Database; ROC: receiver operating characteristic; DCA: decision curve analysis; BMI: body mass index; NMBAs: neuromuscular blockades; APS-III score: acute physiology score III

Patients with the high-level stable phenotype had the highest mortality, and those with the low-level stable phenotype had the lowest mortality compared with the other phenotypes in the crude model, the IPTW model based on logistic regression or XGBoost method (Additional file 7: Figs. S6, Additional file 8: Fig. S7, Additional file 9: Fig. S8). A predictive model was created to identify the characteristics of patients with worse prognosis (high-level stable and ascending phenotypes) and better prognosis (descending and low-level stable phenotypes) to predict ICU mortality. Based on LASSO regression analysis, six variables were selected, and a nomogram was plotted to better visualize the predictive model. The AUC of the training and validation datasets were 0.851 (0.827–0.875) and 0.743 (0.709–0.777), respectively (Fig. 3).

### ARDS mediated the relationship between OSI phenotypes and ICU mortality

ARDS was a significant predictor of ICU mortality in patients with IMV in six models (OR_Model5_: 1.456, 95% CI 1.199–1.768; Additional file 4: Table S4). A causal mediation analysis showed that both indirect and direct effects were significantly associated with ICU mortality (OR_indirect_: 1.027, 95% CI 1.016–1.041; OR_direct_: 1.195, 95% CI 1.126–1.297, with 13.36% of the effects mediated; Additional file 10: Fig. S9). However, over 80% of the effects of the OSI trajectories on ICU mortality in patients with IMV, rather than ARDS, remained unexplained.

### Subgroup analysis

**Fig. 4:**
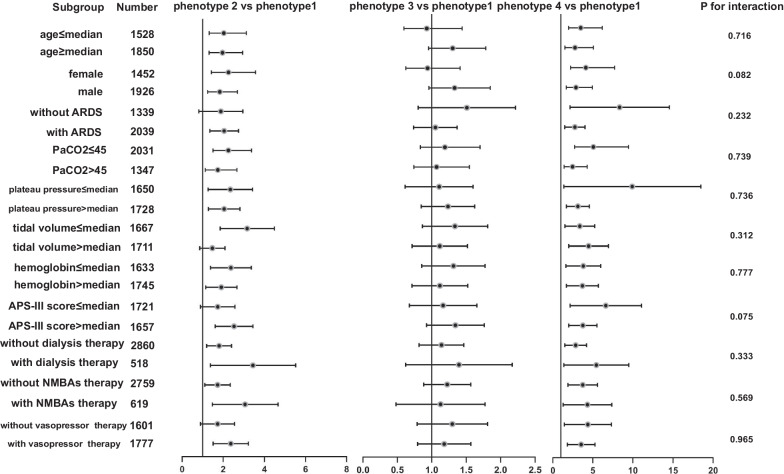
The subgroup analysis of the association between OSI-trajectory based phenotypes and ICU mortality. Almost subgroups showed a significant correlation between the dynamic OSI trajectories and ICU mortality. ARDS: acute respiratory distress syndrome; APS-III score: Acute Physiology III score; NMBAs: neuromuscular blockades; OSI: oxygen saturation index

Various subgroup analyses were performed; the results are presented in Fig. 4 and Additional file 11: Fig. S10. Most subgroups, such as those based on age, plateau pressure, and dialysis therapy, showed a significant correlation between dynamic OSI trajectories and ICU mortality. A predicted marginal effect analysis is shown in Additional file 12: Fig. S11. Patients with ARDS with the ascending and high-level stable phenotypes had higher mortality rates, but those with the descending and low-level stable phenotypes had similar mortality rates compared with patients without ARDS.

## Discussion

To the best of our knowledge, this study was the first to demonstrate an association of dynamic OSI trajectories with ICU mortality in adult patients receiving IMV. The present study had three major findings. First, four different phenotypes of the OSI trajectories were identified, and their association with ICU mortality was evaluated. Patients in the ICU with consistently high OSI values during the first 5 days of hospitalization tended to have higher mortality than those with consistently low OSI values using DR estimation. Second, the prognostic value of the OSI trajectories was validated, and the predictive model could help identify patients with worse prognoses in advance. Third, the effects of the OSI phenotypes on ICU mortality were partially mediated by ARDS.

The present study revealed heterogeneity among patients with mechanical ventilation through the formation of four distinct phenotypes based on dynamically monitored OSI values. In the present study, patients with the high-level stable or ascending phenotype were more likely to have a worse prognosis than those with the other phenotypes. Due to the retrospective nature of this study, determining the exact mechanisms underlying the observed results was difficult. However, patients with the high-level stable or ascending phenotype were more likely to experience hypoxemia and ventilator-induced lung injury (VILI), leading to higher mortality. The OSI was a composite index integrated with the MAP, FiO_2_ and SaO_2_ that can potentially reflect the severity of VILI by measuring oxygen function and ventilation status. The MAP represented the mean alveolar pressure during both the inspiratory and expiratory cycles. High MAP could result in the overinflation of ventilated lung areas and the collapse of dead space, thus increasing mortality [61, 62]. In addition, overdistended alveoli caused by a high MAP could also obstruct venous return and may result in right heart dysfunction through cardiopulmonary interactions, which was consistent with a previous study [63–65]. The FiO_2_/SpO_2_ ratio, a noninvasive indicator, is a good substitute for the oxygenation index (PaO_2_/FiO_2_ ratio) and can effectively identify critically ill patients in advance [66–70]. Therefore, a lower OSI value could potentially reduce VILI. The present data also supported this finding, indicating that the association between OSI values and mortality was mediated by ARDS. Critically ill patients with ARDS experienced a decline in pulmonary compliance and an increase in nonaerated compartments, potentially making them more susceptible to VILI [71, 72]. Previous studies have also demonstrated that ARDS patients with higher OSI values exhibited higher mortality, which was further validated in the present study [5, 20, 73]. These findings confirmed that the OSI-based ventilation strategy allowed for early detection and might potentially facilitate more intensive management of critically ill patients with a poor prognosis.

In the present study, the risk factors for the phenotypes associated with high OSI values were explored and presented in a nomogram that could aid in tailoring the OSI value. For example, PaCO_2_ level at baseline was a predictor for phenotypes with high OSI values. This suggested that patients with hypercapnia are more susceptible to developing VILI, mediated by the OSI values. The potential mechanisms behind this could be attributed to the fact that the blockage or overdistention of alveoli in patients with hypercapnia could cause the instability of pulmonary units and an imbalance in the ventilation/blood flow ratio [74]. Further studies confirmed that hypercapnia was associated with mortality and that extracorporeal CO_2_ removal treatment could correct hypercapnic acidosis, reduce ventilation days and facilitate pulmonary-protective ventilation [42, 75–77]. Therefore, strategies aimed at lowering the OSI value could potentially provide greater benefits to patients with hypercapnia.

In the present study, obesity was another important predictor of high OSI values. This result may partly be explained by the fact that patients with obesity experience more pressure on small airways, resulting in a relatively high prevalence of complete airway closure than patients without obesity (approximately 65% vs 22% in a study by Coudroy et al.) [78, 79]. Complete airway closure could theoretically cause a ventilation perfusion ratio mismatch, accelerate alveolar collapse and impair arterial oxygenation [80]. In addition, Gupta et al. reported that obese patients with IMV treatment were more likely to have higher PEEP and plateau pressure, which might increase MAP values and thus cause high OSI values [81]. Nonetheless, the association between BMI and mortality in critically ill patients with IMV remains controversial. Recently, Ruan et al. reported that BMI was negatively associated with mortality [82]. However, this study included patients using noninvasive ventilation (NIV) or high-flow nasal oxygen therapy (HFNC), who generally had a milder form of the disease compared with the target population in the present study. In contrast, a study by Chetboun et al. showed that BMI was associated with 28-day mortality in critically ill patients with IMV treatment [83]. Consequently, further research is required to investigate the associations between BMI and mortality in critically ill patients receiving IMV.

Our results highlighted the OSI-based dynamic trajectory for the first time. This implied that monitoring a patient’s condition should not be confined solely to the initial 24 h postadmission. Instead, focusing on dynamic changes in relevant indicators is crucial. For example, the initial OSI value of the ascending phenotype was lower than that of the descending phenotype in the present study. However, on the first day of admission, the OSI value of the ascending phenotype surpassed that of the descending phenotype and continued to exhibit this trend throughout the subsequent 4 days of observation. We could speculate that patients with the ascending phenotype had higher mortality than those with the descending phenotype when both patients were indirectly compared with the low-level stable phenotype. Therefore, our study underlined the critical importance of longitudinal surveillance, which can assist clinicians in developing more accurate mechanical ventilation management strategies, potentially resulting in decreased ICU mortality rates.

Our study proposed a simple and user-friendly prediction model of OSI-based phenotypes in evaluating the prognosis for critically ill patients with IMV treatment, which could potentially be of great benefit to assist ICU clinical decisions. This is not only useful in the clarification of the patient's condition to their family prior to the given treatment, but more importantly, it can serve as a prompt biomarker to aid physicians in altering treatment plans during any stage of disease progression to optimize treatment benefits. For example, clinics are more likely to take more active interventions to reduce OSI-induced VILI for phenotypes with a poor prognosis, such as lower driving pressure, low tidal volume ventilation, PEEP titration or extracorporeal CO_2_ removal according to their specific conditions. For ICU patients, whose health condition might change abruptly, this improvement in care strategy could potentially be life-saving. However, further studies are needed to validate their safety and effects.

The present study had some limitations. First, although various statistical method was used to explore the association of OSI-trajectory phenotypes with ICU mortality, the potential confounding effect of several covariables (e.g., plateau pressure, NMBAs) could not be removed entirely. More prospective and rigorously designed studies on this issue are still needed in the future. Second, although the number of patients with the high-level stable phenotype met the criterion of at least 5%, the number of patients in each group was relatively small. Large-scale populations are needed to validate this association in future studies. Third, we were unable to identify the specific reasons leading to IMV due to the design of the database. Therefore, we could not perform subgroup analyses on the causes of IMV (e.g., respiratory, cardiac, and cerebral). Fourth, as this was a retrospective observational study, the association between OSI-trajectory phenotypes and ICU mortality could only be speculated. Therefore, further prospective studies should be conducted to establish cause–effect relationships.

## Conclusions

In our retrospective cohort study, four OSI-trajectory phenotypes were identified. The high-level stable and ascending phenotypes were associated with higher mortality than the low-level stable and descending phenotypes in ICU patients with IMV treatment within the first 5 days after admission. These four phenotypes could help identify patients with poor prognoses in advance, providing valuable insights for clinical practice.

### Supplementary Information

**Additional file 1: Figure S1.** The percentage of missing values among variables. PEEP: positive end expiratory pressure; MIMIC-IV: the Medical Information Market for Intensive Care IV; EICU-CRD: the eICU Collaborative Research Database.

**Additional file 2: Figure S2.** The DAGs showed covariates of the association between the OSI-based trajectory phenotypes and ICU mortality. The demographics included age, gender and ethnicity. The respiratory comorbidities included ARDS, COPD. Other comorbidities included CKD, AKI, malignancy, HF and diabetes. PaCO_2_ and hemoglobin were included in the laboratory events. The ventilation parameters included PEEP, plateau pressure, tidal volume, OSI at baseline and the treatment included dialysis, NMBAs and vasopressor therapy. DAGs: Directed acyclic graphs; BMI: body mass index; OSI: oxygen saturation index; PEEP: positive end expiratory pressure; NMBAs: neuromuscular blockades; APS-III score: acute physiology score III; ARDS: acute respiratory distress syndrome; AKI: acute kidney injury; COPD: chronic obstructive pulmonary disease; HF: heart failure; CKD: chronic kidney disease.

**Additional file 3: Figure S3.** Flowchart of patient enrollment and statistical analysis. After application of exclusion criteria, a total of 3378 patients were included into further analysis. The best number of phenotypes were determined first by GBTM method. After comparison of characteristics of each phenotype with standard statistical method, the association of OSI-trajectory phenotypes with ICU mortality were explored using DR estimation. After that, the risk factors of OSI-trajectory phenotypes with a poor prognosis were determined by the SMOTE algorithm and predictive model. CMA analysis was used to determine the casual mediation effect of ARDS on the prognostic value of OSI-trajectory phenotypes. Subgroup analysis and predictive marginal effect analysis was used to validate the robustness of prognostic value of OSI-trajectory phenotypes. Kaplan–Meier analysis was used to explore the prognostic value of OSI-trajectory phenotypes on long-term outcomes. MIMIC-IV: the Medical Information Market for Intensive Care IV; EICU-CRD: the eICU Collaborative Research Database; OSI: oxygen saturation index; CMA: causal mediation analysis; ARDS: acute respiratory distress syndrome; GBTM: group based trajectory model; MAP: mean airway pressure; SMOTE: synthetic minority oversampling technique.

**Additional file 4: Table S1.** Results of group-based trajectory modeling. **Table S2.** The analysis of optimal trend for trajectories. **Table S3.** Results of doubly robust estimation of two databases. **Table S4.** The associations between ARDS and ICU mortality.

**Additional file 5: Figure S4.** The loveplot of covariables. The SMD generated by IPTW (logistic) or IPTW (Xgboost) were significantly smaller than the SMD in crude covariables. SMD: standardized mean difference; OSI: oxygen saturation index; PEEP: positive end expiratory pressure; NMBAs: neuromuscular blockades; APS: acute physiology score III; ARDS: acute respiratory distress syndrome; AKI: acute kidney injury; BMI: body mass index; HB: hemoglobin; COPD: chronic obstructive pulmonary disease; HF: heart failure; CKD: chronic kidney disease.

**Additional file 6: Figure S5.** The comparison of ICU mortality (a) and VFDs (b) among four phenotypes in two datasets. IPTW: inverse probability of treatment weighting; MIMIC-IV: the Medical Information Market for Intensive Care IV; EICU-CRD: the eICU Collaborative Research Database.

**Additional file 7: Figure S6. **The Kaplan–Meier survival curve of 21d-mortality in crude data. The survival rate of patients in group 4 (high-level stable) were significantly lower than other groups.

**Additional file 8: Figure S7.** The Kaplan–Meier survival curve of 21d-mortality using IPTW method by multinomial logistic regression. The survival rate of patients in group 4 (high-level stable) were significantly lower than other groups.

**Additional file 9: Figure S8.** The Kaplan–Meier survival curve of 21d-mortality using IPTW method by Xgboost. The survival rate of patients in group 4 (high-level stable) were significantly lower than other groups.

**Additional file 10: Figure S9.** ARDS mediates about 13.36% effect of OSI trajectory index on ICU mortality. OSI: oxygen saturation index.

**Additional file 11: Figure S10.** The subgroup analysis of the association between OSI-trajectory based phenotypes and ICU mortality. COPD: chronic obstructive pulmonary disease; CKD: chronic kidney disease; AKI: acute kidney injury

**Additional file 12: Figure S11.** The predictive marginal effect of ARDS on ICU mortality in patients with or without ARDS. ARDS: acute respiratory distress syndrome; OSI: oxygen saturation index.

## Data Availability

All data used in our study could be available in the MIMIC-IV (https://physionet.org/content/mimiciv/2.0/) and EICU-CRD database (https://physionet.org/content/eicu-crd/2.0/).

## Abbreviations

ACME: Average causal mediation effect
ADE: Average direct effect
APS-III: Acute Physiology III score
ARDS: Acute respiratory distress syndrome
AUC: Area under the curve
BIC: Bayesian information criterion
BMI: Body mass index
CI: Confidence interval
eICU-CRD: EICU Collaborative Research Database
GBTM: Group-based trajectory model
HFNC: High-flow nasal cannula
ICU: Intensive care unit
IMV: Invasive mechanical ventilation
IPTW: Inverse probability of treatment weighting
LASSO: Least Absolute Shrinkage and Selection Operator
MAP: Mean airway pressure
MIMIC-IV: Medical Information Market for Intensive Care IV
NMBAs: Neuromuscular blockades
OI: Oxygen index
OR: Odds ratio
OSI: Oxygen saturation index
PEEP: Positive end-expiratory pressure
SMD: Standardized mean difference
SMOTE: Synthetic Minority Over-sampling Technique
SOFA: Sequential Organ Failure Assessment score
VFDs: Ventilation-free days
XGBoost: Extreme Gradient Boosting
