# Supplementary figures and images for: Prognostic value of oxygen saturation index trajectory phenotypes on ICU mortality in mechanically ventilated patients: a multi-database retrospective cohort study
Source: J Intensive Care. 2023 Nov 29;11:59. doi: 10.1186/s40560-023-00707-x (PMC10685672; doi:10.1186/s40560-023-00707-x)

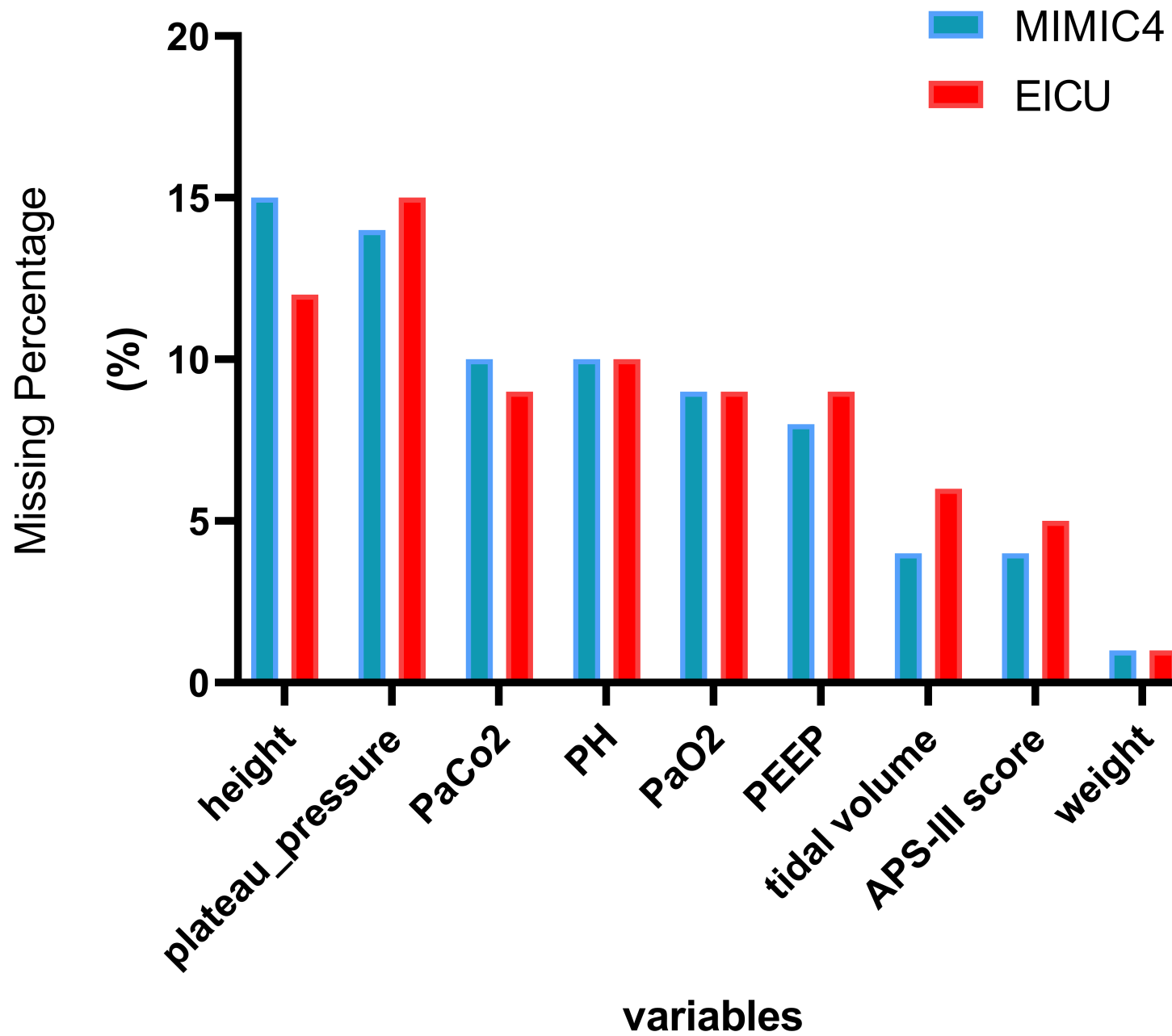

Supplement: Supplementary file 1 — Additional file 1: Figure S1. The percentage of missing values among variables. PEEP: positive end expiratory pressure; MIMIC-IV: the Medical Information Market for Intensive Care IV; EICU-CRD: the eICU Collaborative Research Database. [file 40560_2023_707_MOESM1_ESM.pdf]

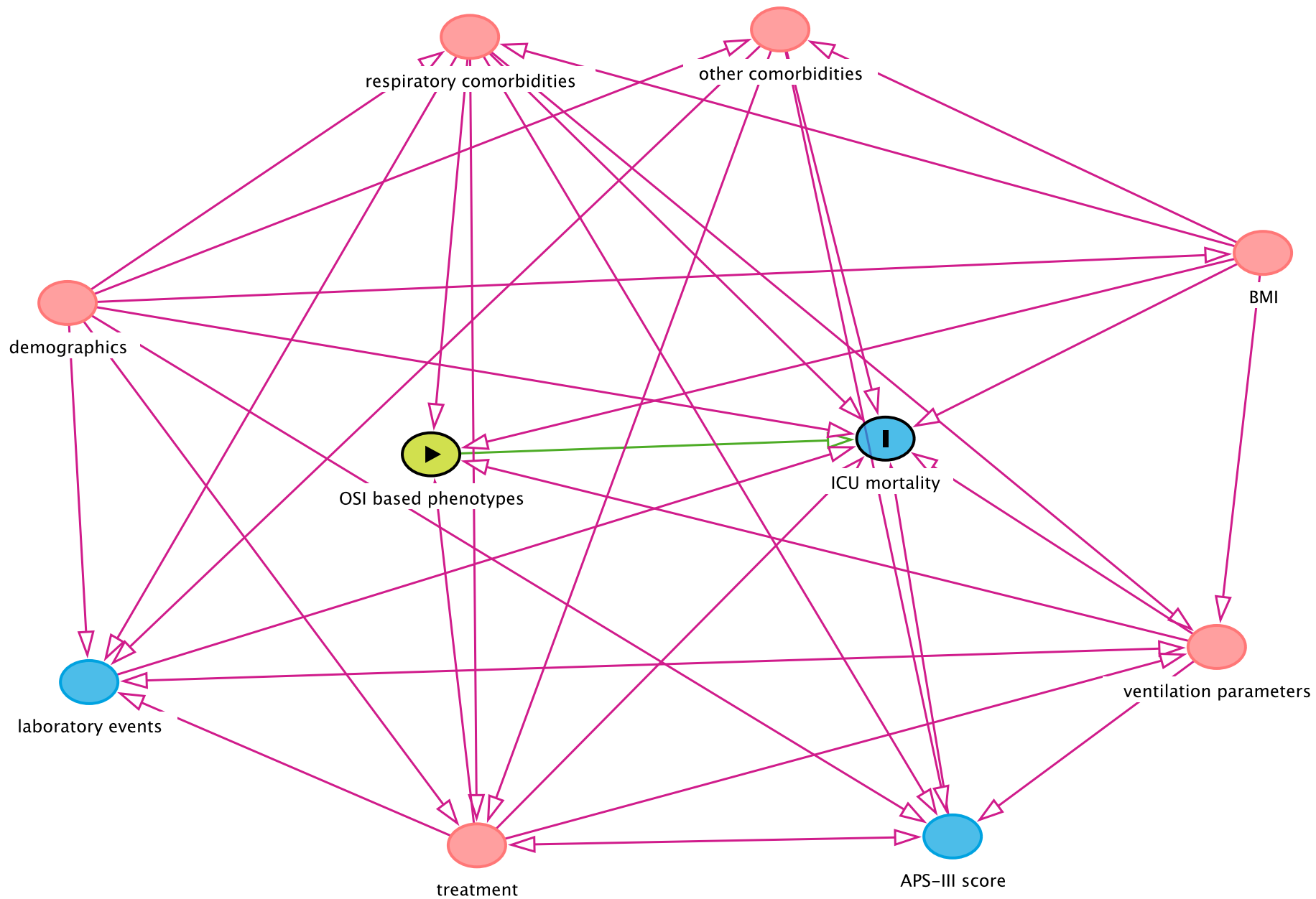

Supplement: Supplementary file 2 — Additional file 2: Figure S2. The DAGs showed covariates of the association between the OSI-based trajectory phenotypes and ICU mortality. The demographics included age, gender and ethnicity. The respiratory comorbidities included ARDS, COPD. Other comorbidities included CKD, AKI, malignancy, HF and diabetes. PaCO2 and hemoglobin were included in the laboratory events. The ventilation parameters included PEEP, plateau pressure, tidal volume, OSI at baseline and the treatment included dialysis, NMBAs and vasopressor therapy. DAGs: Directed acyclic graphs; BMI: body mass index; OSI: oxygen saturation index; PEEP: positive end expiratory pressure; NMBAs: neuromuscular blockades; APS-III score: acute physiology score III; ARDS: acute respiratory distress syndrome; AKI: acute kidney injury; COPD: chronic obstructive pulmonary disease; HF: heart failure; CKD: chronic kidney disease. [file 40560_2023_707_MOESM2_ESM.pdf]

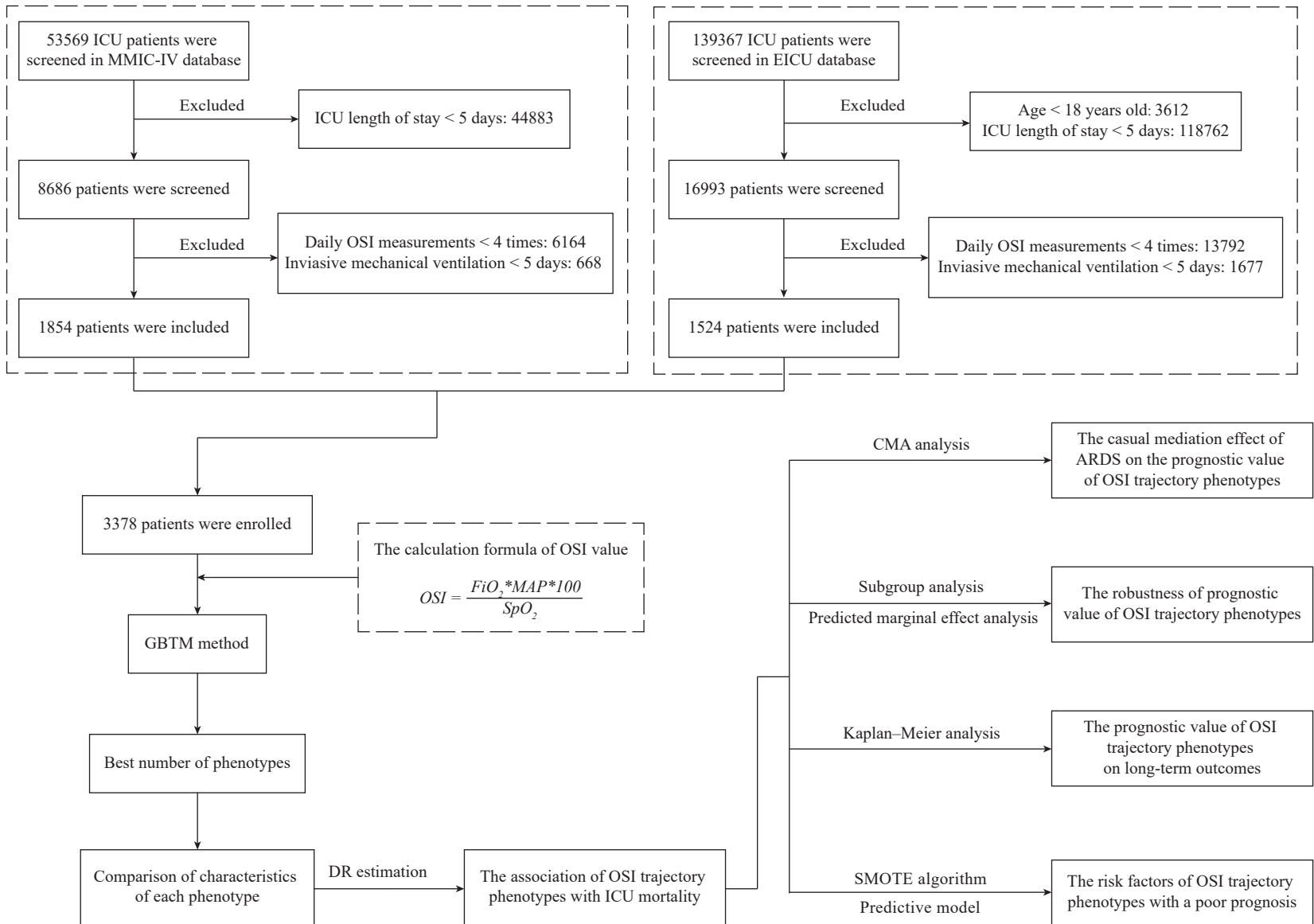

Supplement: Supplementary file 3 — Additional file 3: Figure S3. Flowchart of patient enrollment and statistical analysis. After application of exclusion criteria, a total of 3378 patients were included into further analysis. The best number of phenotypes were determined first by GBTM method. After comparison of characteristics of each phenotype with standard statistical method, the association of OSI-trajectory phenotypes with ICU mortality were explored using DR estimation. After that, the risk factors of OSI-trajectory phenotypes with a poor prognosis were determined by the SMOTE algorithm and predictive model. CMA analysis was used to determine the casual mediation effect of ARDS on the prognostic value of OSI-trajectory phenotypes. Subgroup analysis and predictive marginal effect analysis was used to validate the robustness of prognostic value of OSI-trajectory phenotypes. Kaplan–Meier analysis was used to explore the prognostic value of OSI-trajectory phenotypes on long-term outcomes. MIMIC-IV: the Medical Information Market for Intensive Care IV; EICU-CRD: the eICU Collaborative Research Database; OSI: oxygen saturation index; CMA: causal mediation analysis; ARDS: acute respiratory distress syndrome; GBTM: group based trajectory model; MAP: mean airway pressure; SMOTE: synthetic minority oversampling technique. [file 40560_2023_707_MOESM3_ESM.pdf]

variable

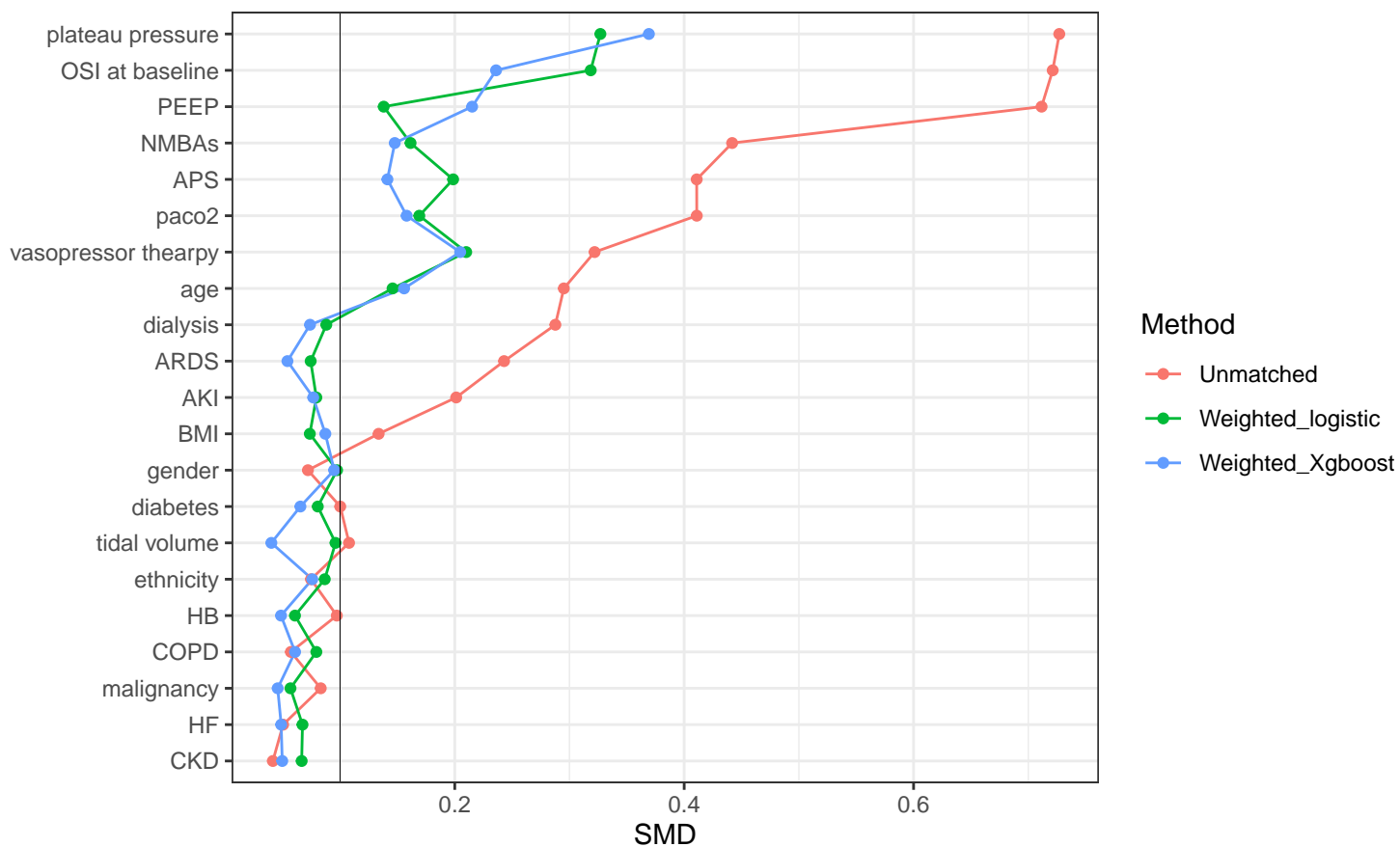

Supplement: Supplementary file 5 — Additional file 5: Figure S4. The loveplot of covariables. The SMD generated by IPTW (logistic) or IPTW (Xgboost) were significantly smaller than the SMD in crude covariables. SMD: standardized mean difference; OSI: oxygen saturation index; PEEP: positive end expiratory pressure; NMBAs: neuromuscular blockades; APS: acute physiology score III; ARDS: acute respiratory distress syndrome; AKI: acute kidney injury; BMI: body mass index; HB: hemoglobin; COPD: chronic obstructive pulmonary disease; HF: heart failure; CKD: chronic kidney disease. [file 40560_2023_707_MOESM5_ESM.pdf]

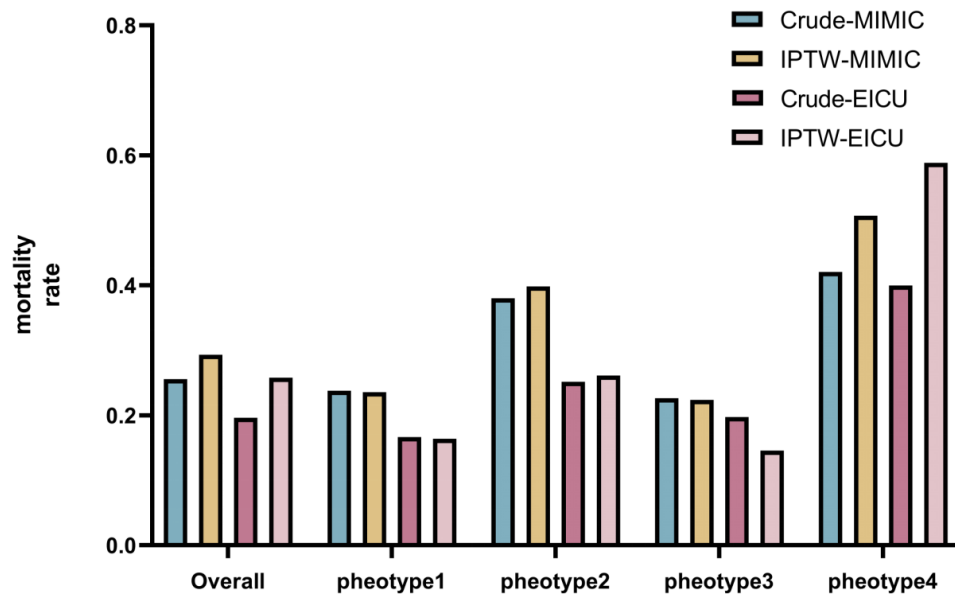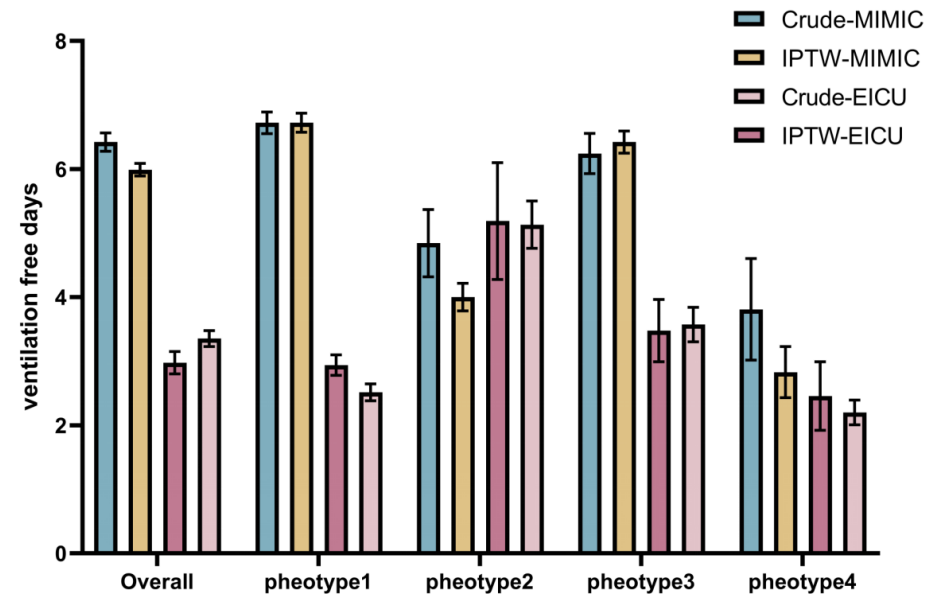

Supplement: Supplementary file 6 — Additional file 6: Figure S5. The comparison of ICU mortality (a) and VFDs (b) among four phenotypes in two datasets. IPTW: inverse probability of treatment weighting; MIMIC-IV: the Medical Information Market for Intensive Care IV; EICU-CRD: the eICU Collaborative Research Database. [file 40560_2023_707_MOESM6_ESM.pdf]

Kaplan–Meier survival estimates

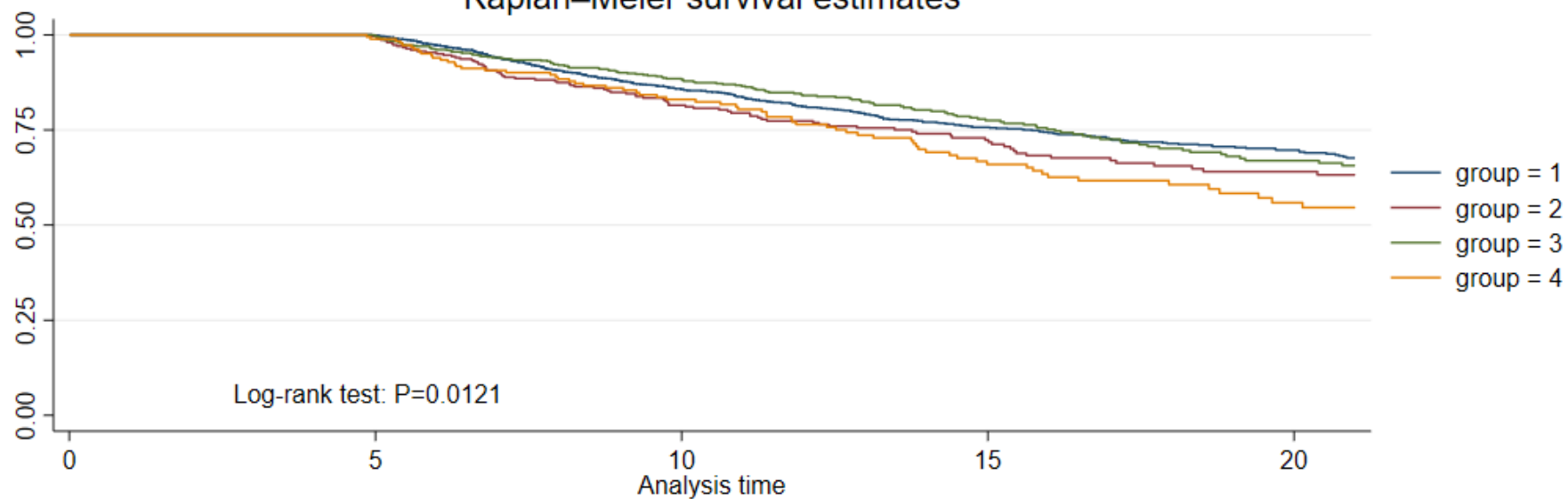

Number at risk

|           |      |      |      |     |     |
|-----------|------|------|------|-----|-----|
| group = 1 | 2194 | 2178 | 1222 | 575 | 288 |
| group = 2 | 301  | 300  | 208  | 127 | 76  |
| group = 3 | 700  | 697  | 422  | 212 | 107 |
| group = 4 | 183  | 181  | 131  | 81  | 45  |

Supplement: Supplementary file 7 — Additional file 7: Figure S6. The Kaplan–Meier survival curve of 21d-mortality in crude data. The survival rate of patients in group 4 (high-level stable) were significantly lower than other groups. [file 40560_2023_707_MOESM7_ESM.pdf]

Kaplan–Meier survival estimates

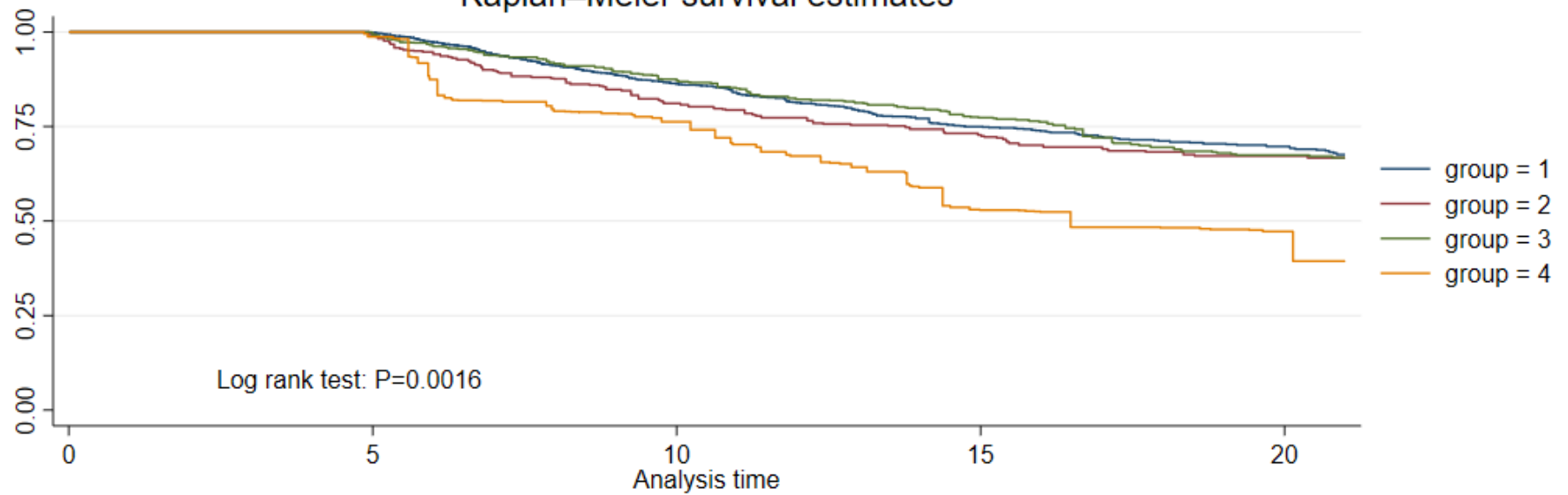

Number at risk

|           |          |          |          |          |          |
|-----------|----------|----------|----------|----------|----------|
| group = 1 | 3058.439 | 3038.839 | 1744.385 | 824.3666 | 414.2928 |
| group = 2 | 2200.439 | 2186.396 | 1466.386 | 915.9443 | 524.5013 |
| group = 3 | 2351.723 | 2333.77  | 1334.702 | 701.1558 | 353.0979 |
| group = 4 | 1707.667 | 1685.852 | 1052.874 | 584.5459 | 293.6305 |

Supplement: Supplementary file 8 — Additional file 8: Figure S7. The Kaplan–Meier survival curve of 21d-mortality using IPTW method by multinomial logistic regression. The survival rate of patients in group 4 (high-level stable) were significantly lower than other groups. [file 40560_2023_707_MOESM8_ESM.pdf]

Kaplan–Meier survival estimates

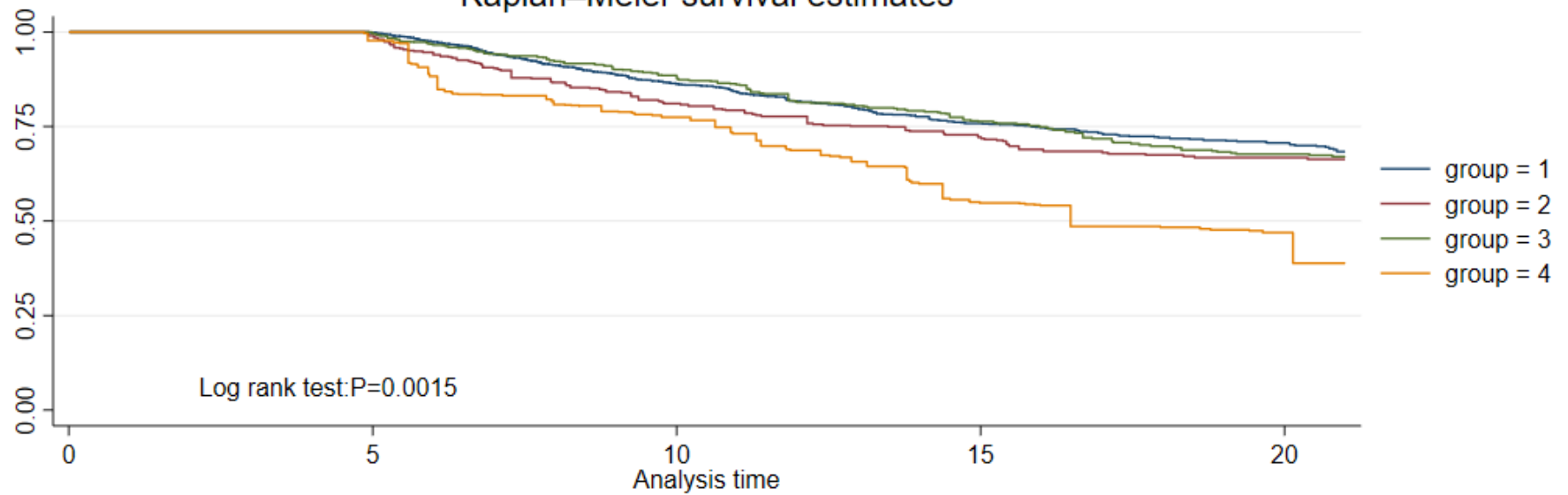

Number at risk

|           |          |          |          |          |          |
|-----------|----------|----------|----------|----------|----------|
| group = 1 | 3061.428 | 3041.988 | 1752.486 | 832.7009 | 423.7398 |
| group = 2 | 1657.52  | 1640.969 | 1133.296 | 683.9152 | 380.6523 |
| group = 3 | 2368.711 | 2356.577 | 1351.973 | 680.5628 | 348.8592 |
| group = 4 | 1257.847 | 1229.085 | 797.2381 | 447.4154 | 196.0448 |

Supplement: Supplementary file 9 — Additional file 9: Figure S8. The Kaplan–Meier survival curve of 21d-mortality using IPTW method by Xgboost. The survival rate of patients in group 4 (high-level stable) were significantly lower than other groups. [file 40560_2023_707_MOESM9_ESM.pdf]

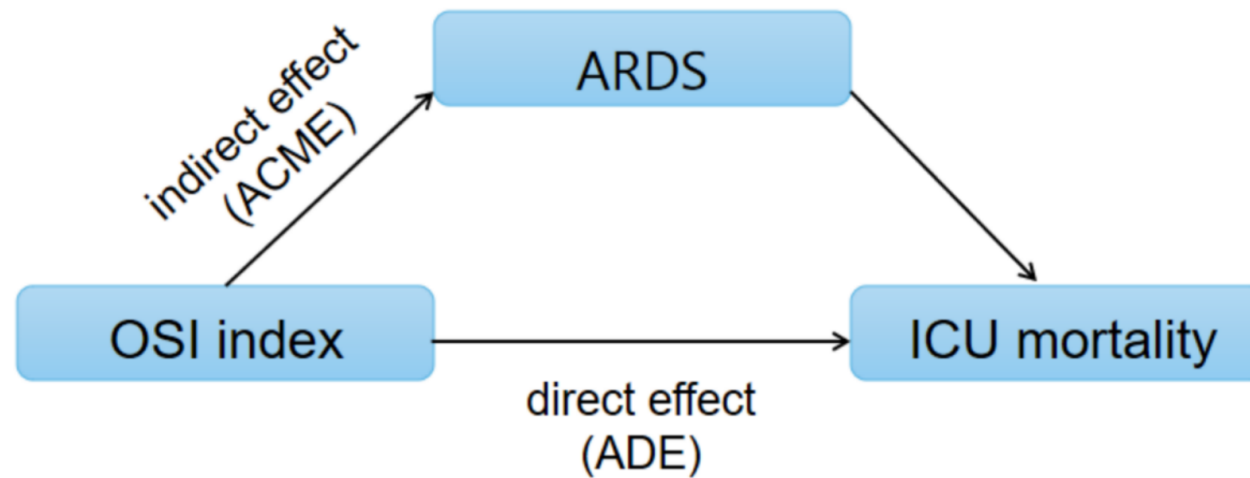

| Average direct effect |               |       | Average causal mediate effect |               |       | M%     |
|-----------------------|---------------|-------|-------------------------------|---------------|-------|--------|
| OR                    | 95% CI        | P     | OR                            | 95% CI        | P     |        |
| 1.195                 | (1.126-1.297) | <0.01 | 1.027                         | (1.016-1.041) | <0.01 | 13.36% |

Supplement: Supplementary file 10 — Additional file 10: Figure S9. ARDS mediates about 13.36% effect of OSI trajectory index on ICU mortality. OSI: oxygen saturation index. [file 40560_2023_707_MOESM10_ESM.pdf]

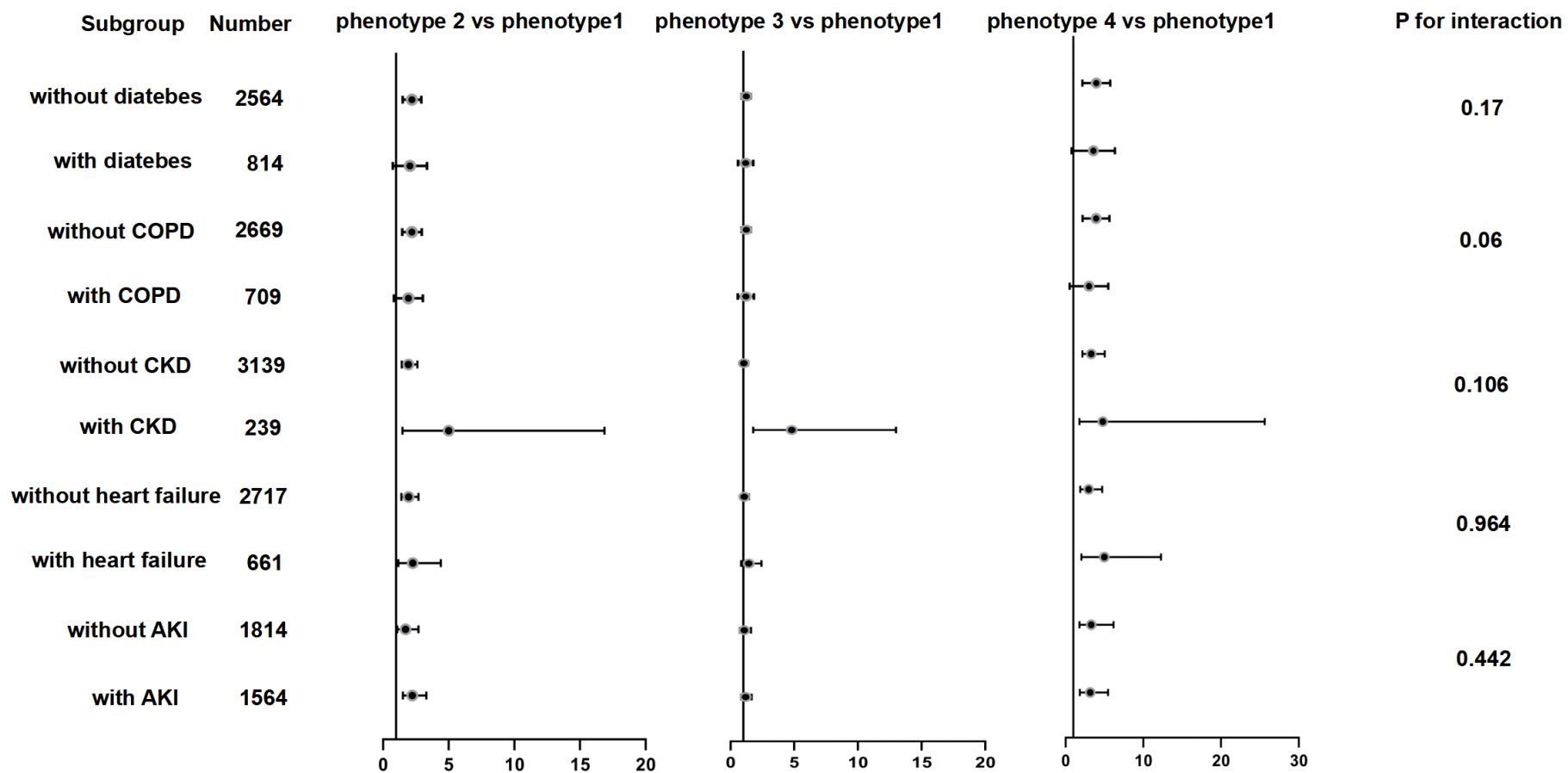

Supplement: Supplementary file 11 — Additional file 11: Figure S10. The subgroup analysis of the association between OSI-trajectory based phenotypes and ICU mortality. COPD: chronic obstructive pulmonary disease; CKD: chronic kidney disease; AKI: acute kidney injury [file 40560_2023_707_MOESM11_ESM.pdf]

Predicted marginal effect of ARDS

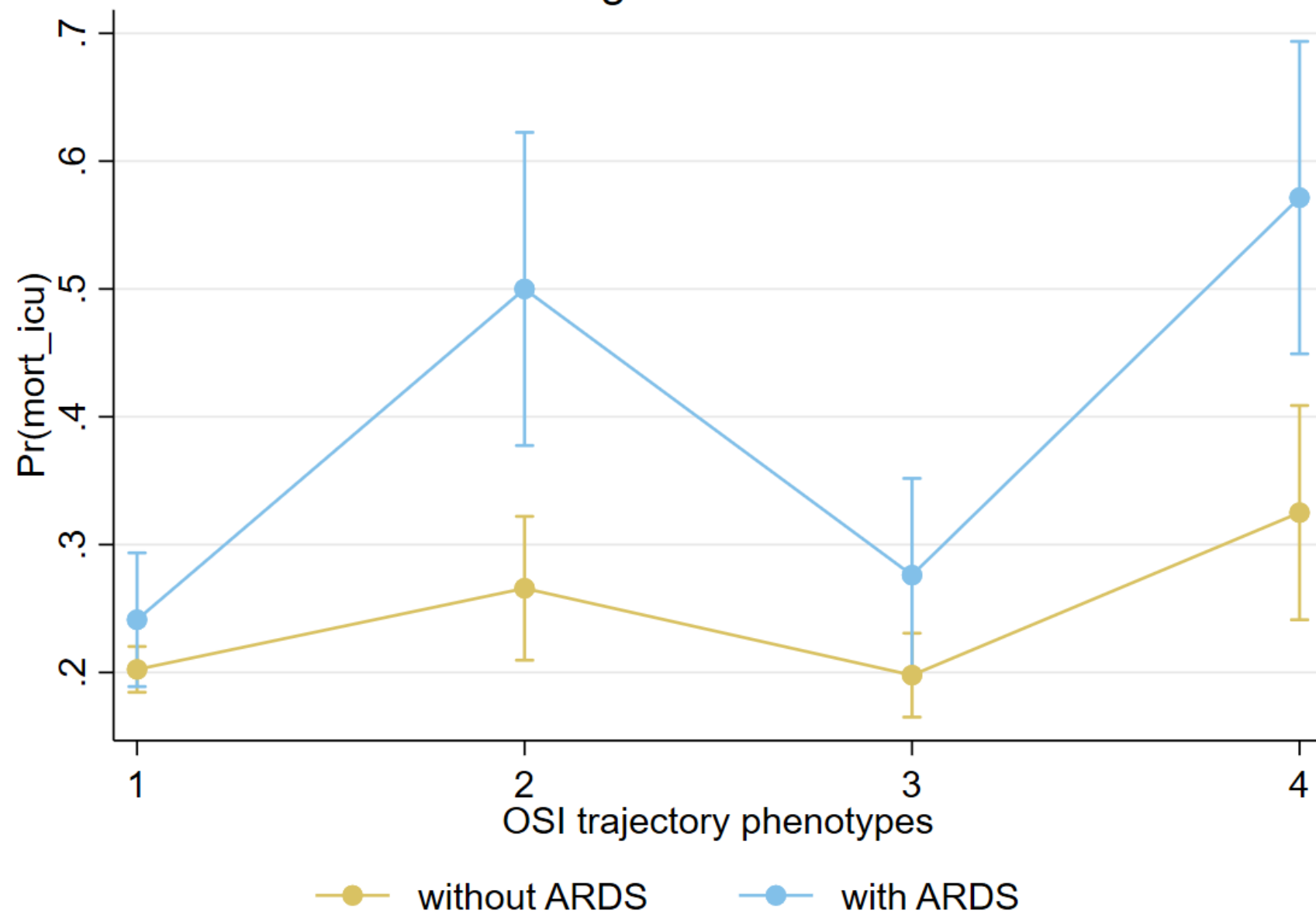

Supplement: Supplementary file 12 — Additional file 12: Figure S11. The predictive marginal effect of ARDS on ICU mortality in patients with or without ARDS. ARDS: acute respiratory distress syndrome; OSI: oxygen saturation index. [file 40560_2023_707_MOESM12_ESM.pdf]
