# Supplementary material for: Prognostic value of oxygen saturation index trajectory phenotypes on ICU mortality in mechanically ventilated patients: a multi-database retrospective cohort study
Source: J Intensive Care. 2023 Nov 29;11:59. doi: 10.1186/s40560-023-00707-x (PMC10685672; doi:10.1186/s40560-023-00707-x)
Supplement: Supplementary file 4 — Additional file 4: Table S1. Results of group-based trajectory modeling. Table S2. The analysis of optimal trend for trajectories. Table S3. Results of doubly robust estimation of two databases. Table S4. The associations between ARDS and ICU mortality. [file 40560_2023_707_MOESM4_ESM.docx]

Table S1 Results of group-based trajectory modeling.

| Number of  groups | Trajectory  shapes | Log-likelihood | Akaike information criterion | Bayesian  Information  Criterion | Participants per group (%) | Mean posterior probabilities | Entropy |
| --- | --- | --- | --- | --- | --- | --- | --- |

| 2 | Linear | -175095.24 | -175101.24 | -175119.61 | 82.776/17.224 | 0.997/0.986 | 0.983 |
| --- | --- | --- | --- | --- | --- | --- | --- |
|  | Quadratic | -174752.64 | -174760.64 | -174785.14 | 82.781/17.219 | 0.998/0.986 | 0.984 |
|  | Cubic | -174584.32 | -174594.32 | -174624.94 | 82.786/17.214 | 0.997/0.986 | 0.984 |
| 3 | Linear | -168185.66 | -168194.66 | -168222.22 | 66.600/26.266/7.133 | 0.990/0.976/0.989 | 0.969 |
|  | Quadratic | -167658.66 | -167670.66 | -167707.41 | 66.093/26.429/7.478 | 0.991/0.972/0.992 | 0.969 |
|  | Cubic | -167423.00 | -167438.00 | -167483.94 | 66.353/26.379/7.268 | 0.990/0.975/0.992 | 0.969 |
| 4 | Linear | -165050.05 | -165062.05 | -165098.80 | 56.825/28.717/11.048/3.410 | 0.980/0.955/0.984/0.995 | 0.953 |
|  | Quadratic | -164447.26 | -164463.26 | -164512.26 | 56.733/28.830/11.049/3.388 | 0.982/0.954/0.981/0.998 | 0.953 |
|  | Cubic | -164763.60 | -164783.60 | -164844.85 | 9.122/64.717/20.786/5.375 | 0.961/0.991/0.956/0.992 | 0.967 |

According to the statistical model, the number of trajectories that provided the lowest -2*log-likelihood, AIC, and BIC values was determined to be 4. Therefore, this was deemed as the most suitable number of trajectories.

AIC: Akaike information criterion; BIC: Bayesian information criteria.

Table S2 . The analysis of optimal trend for trajectories.

| Trends of each  Trajectories^#^ | Group | Parameter | Estimate | Standard Error | T for H0^*^ | Prob > \|T\| |
| --- | --- | --- | --- | --- | --- | --- |
| 3 3 3 3 | 1 | Intercept | 9.91439 | 0.32816 | 30.212 | 0.0000 |
|  |  | Linear | -0.62132 | 0.07448 | -8.342 | 0.0000 |
|  |  | Quadratic | 0.09425 | 0.00901 | 10.455 | 0.0000 |
|  |  | Cubic | -0.00309 | 0.00028 | -10.948 | 0.0000 |
|  | 2 | Intercept | 6.28289 | 0.07556 | 83.146 | 0.0000 |
|  |  | Linear | -0.63942 | 0.02584 | -24.749 | 0.0000 |
|  |  | Quadratic | 0.05364 | 0.00278 | 19.319 | 0.0000 |
|  |  | Cubic | -0.00140 | 0.00009 | -16.222 | 0.0000 |
|  | 3 | Intercept | 14.05893 | 0.23863 | 58.915 | 0.0000 |
|  |  | Linear | -1.32654 | 0.05359 | -24.753 | 0.0000 |
|  |  | Quadratic | 0.08126 | 0.00543 | 14.960 | 0.0000 |
|  |  | Cubic | -0.00182 | 0.00017 | -10.992 | 0.0000 |
|  | 4 | Intercept | 16.93788 | 0.22865 | 74.077 | 0.0000 |
|  |  | Linear | 0.43487 | 0.08863 | 4.907 | 0.0000 |
|  |  | Quadratic | -0.06090 | 0.00962 | -6.328 | 0.0000 |
|  |  | Cubic | 0.00164 | 0.00030 | 5.449 | 0.0000 |
| 2 2 2 2 | 1 | Intercept | 5.33118 | 0.05110 | 104.328 | 0.0000 |
|  |  | Linear | -0.27013 | 0.01037 | -26.050 | 0.0000 |
|  |  | Quadratic | 0.00992 | 0.00047 | 21.050 | 0.0000 |
|  | 2 | Intercept | 9.77030 | 0.08254 | 118.370 | 0.0000 |
|  |  | Linear | -0.46689 | 0.01583 | -29.502 | 0.0000 |
|  |  | Quadratic | 0.01506 | 0.00070 | 21.444 | 0.0000 |
|  | 3 | Intercept | 14.59527 | 0.12089 | 120.728 | 0.0000 |
|  |  | Linear | -0.43273 | 0.02461 | -17.584 | 0.0000 |
|  |  | Quadratic | 0.00946 | 0.00111 | 8.559 | 0.0000 |
|  | 4 | Intercept | 16.60257 | 0.19143 | 86.730 | 0.0000 |
|  |  | Linear | 0.41949 | 0.04188 | 10.018 | 0.0000 |
|  |  | Quadratic | -0.02136 | 0.00193 | -11.087 | 0.0000 |

#: Each number represents the type of trend for a corresponding trajectory. For example, in a model with a trend sequence of "1 2 3," the value "1" indicates a linear trend for the first trajectory, "2" suggests using a quadratic trend for the second trajectory, while "3" indicates that a cubic trend should be used for the final trajectory.

*: H0 : parameter = 0

Table S3 Results of doubly robust estimation of two databases *

|  | Phenotype 1 | Phenotype 2 |  | Phenotype 3 |  | Phenotype 4 |  |
| --- | --- | --- | --- | --- | --- | --- | --- |
|  |  | OR(95%CI) | P value | OR(95%CI) | P value | OR(95%CI) | P value |
| **MIMIC-IV** |  |  |  |  |  |  |  |
| Propensity score IPTW | Reference | 1.692(1.277-2.241) | <0.001 | 0.951(0.722-1.252) | 0.718 | 2.153(1.303-3.557) | 0.003 |
| Doubly robust with unbalanced covariables | Reference | 1.730(1.306-2.291) | <0.001 | 0.967(0.736-1.271) | 0.811 | 1.878(1.314-2.683) | <0.001 |
| Doubly robust with all covariables | Reference | 1.637(1.252-2.141) | <0.001 | 0.931(0.711-1.220) | 0.603 | 1.775(1.322-2.382) | <0.001 |
|  |  |  |  |  |  |  |  |
| **EICU** |  |  |  |  |  |  |  |
| Propensity score IPTW | Reference | 1.591(1.091-2.321) | 0.016 | 0.888(0.637-1.238) | 0.484 | 3.588(2.538-5.071) | <0.001 |
| Doubly robust with unbalanced covariables | Reference | 1.593(1.088-2.334) | 0.017 | 0.891(0.634-1.252) | 0.506 | 3.606(2.484-5.235) | <0.001 |
| Doubly robust with all covariables | Reference | 1.598(1.117-2.285) | 0.010 | 0.872(0.620-1.228) | 0.433 | 3.687(2.438-5.577) | <0.001 |

*Note:The IPTW method was based on XGBoost algorithm.

Table S4 The associations between ARDS and ICU mortality

OR 95%CI P

Model_0_  2.018 (1.692-2.406) ‹0.001

Model_1_ 1.916 (1.603-2.290) ‹0.001

Model_2_ 1.590 (1.321-1.914) ‹0.001

Model_3_ 1.563 (1.295-1.886) ‹0.001

Model_4_ 1.498 (1.237-1.814) ‹0.001

Model_5_ 1.456 (1.199-1.768) ‹0.001

Model_0_: univariate model without adjustment of covariates.

Model_1_: adjusted for age, gender, ethnicity.

Model_2_: additionally adjusted for APS-III score, OSI at baseline, BMI, paco2, hemoglobin on model 1.

Model_3_: additionally adjusted for PEEP, plateau pressure, tidal volume upon model 2.

Model_4_: additionally adjusted for differential comorbidities upon model 3.

Model_5_: additionally adjusted for dialysis, vasopressors, NMBAs upon model 4.

OSI, oxygen saturation index; APS-III score, acute physiology III score ; BMI, body mass index; PEEP, positive end expiratory pressure; NMBAs, neuromuscular blockades; ARDS, acute respiratory distress syndrome; ICU, intensive care unit
